# Supplementary material for: Epidemiology of revision hip replacement surgery in the UK over the past 15 years—an analysis from the National Joint Registry
Source: BMJ Open. 2023 Oct 17;13(10):e072462. doi: 10.1136/bmjopen-2023-072462 (PMC10583040; doi:10.1136/bmjopen-2023-072462)
Supplement: Supplementary data [file bmjopen-2023-072462supp001.pdf]

Supplementary Data

Appendix A - Methodology for calculation of directly standardised rates (DSRs) for pHR and rHR

Population estimates

The description that follows of the methodology for calculating directly standardised rates is reproduced from our recent publication [1].

We used mid-year population estimates from the Office for National Statistics (ONS) to calculate changes in the usual resident population. We filtered out population data for Scotland because it has its own register and does not report to the NJR. For simplicity, we have continued to refer to the data remaining (for England, Wales and Northern Ireland) as the 'UK population'. In addition, we have not accounted for population estimates for the Isle of Man and the States of Guernsey, which recently joined the NJR.

The total UK population increased in size every year over the study period, with overall growth of 10.1% from mid-2006 (55693967 persons) to mid-2019 (61333507 persons) [2]. The adult population (i.e. persons aged 18 years or older) increased by 10.9% from mid-2006 (43511312 persons) to mid-2019 (48239295 persons).

Methodology for population standardisation

We performed direct standardisation using Byar's method with Dobson method adjustment following Public Health England guidance [3]. The phe_dsr function within the PHEindicatormethods package in R was used. The 2013 European Standard Population (ESP) was chosen as the standard population. It is a theoretical population created from European Union states' population projections for 2011 - 2030. The population is divided into five-year age groups, starting from 0-4 years until 90 years and older. Since nearly all pHR and rHR are performed in adult patients, we truncated and recategorised the European Standard Population to match the age grouped we defined for the NJR (i.e. 18-49 years, 50-59 years, 60-69 years, 70-79 years, and 80+ years). To calculate the size of the 18-59 years age group, we calculated the total of all age groups from 20-24 years to 45-49 years, and then added two-fifths of the total of the 15-19 years age group. Finally, the resulting vector was transformed to provide a standard population of 100,000 persons.

Trends over time in directly standardised rates of pHR and rHR

Directly standardised rates of pHR and rHR are plotted together with crude incidence rates in Appendix A Figure 1. These rates allow other regions (including those with different age structures) to compare their rate of intervention to the UK.

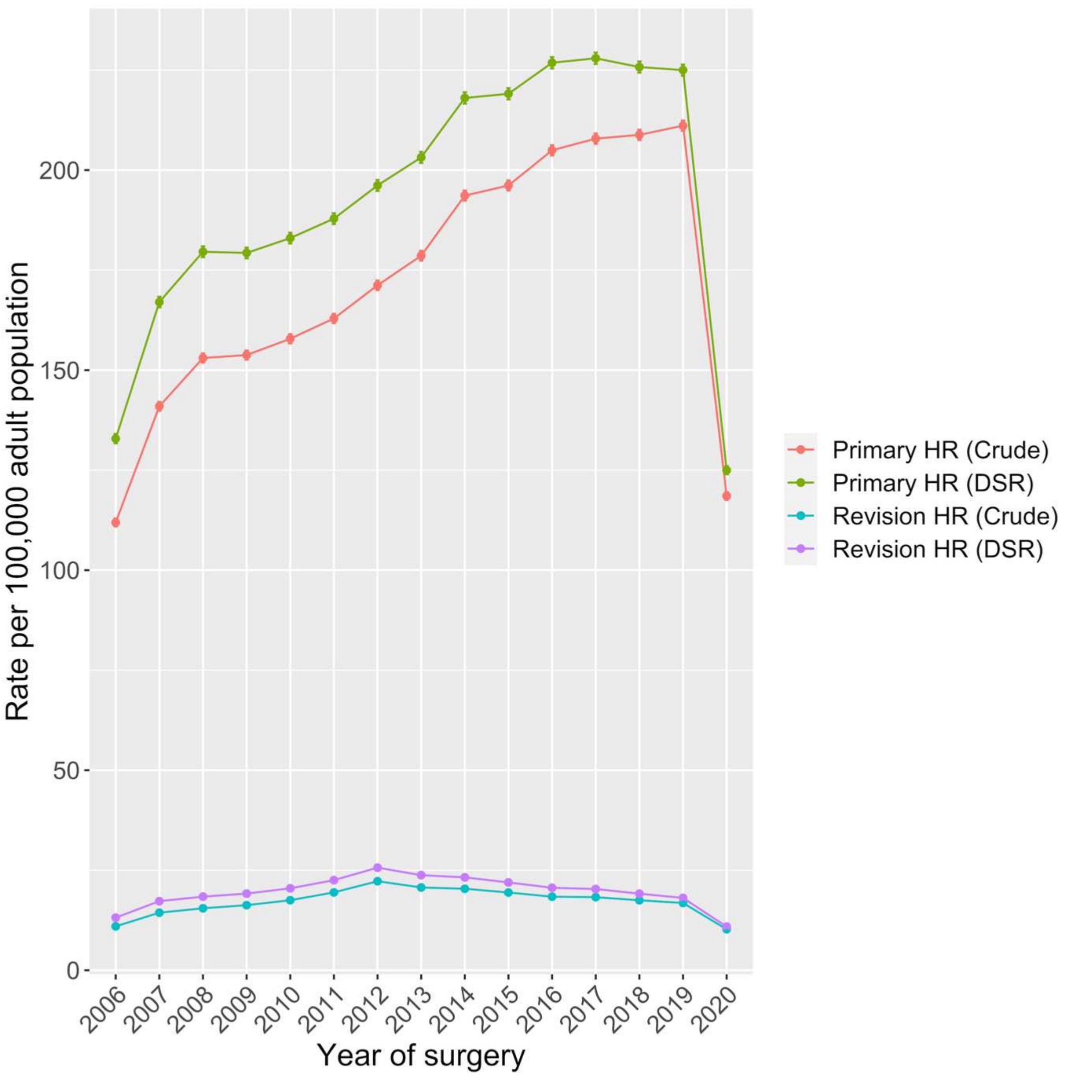

Appendix A Figure 1: Directly standardised rates (DSR) (using the European Standard Population 2013) and crude rates of pHR and rHR from 2006-2020.

Appendix B - Diagnosis hierarchy for rHR

Appendix B Table 1: Diagnosis hierarchy for rHR

Rank	Indication for revision	NJR indication(s) included	AOANJRR category
1	Infection	Infection	Dominant diagnosis independent of prosthesis/surgery
2	Malalignment/Size mismatch	Head/Socket Mismatch<br>Malalignment	Surgical procedure
3*	Adverse soft tissue reaction	Adverse Soft Tissue Reaction to Particulate Debris	Reaction to prosthesis
4	Loosening/Lysis	Aseptic loosening<br>Lysis	Reaction to prosthesis
5	Component wear/Breakage	Implant fracture<br>Wear of acetabular component	Wear and implant breakage
6	Dislocation/Instability	Dislocation/Subluxation<br>Dissocation of Liner	Stability of prosthesis
7	Fracture	Peri-Prosthetic Fracture	Fracture of bone
9	Unexplained pain	Unexplained pain	Pain
10	Other	Other	Remaining diagnoses

*Indication added to NJR in 2008

Appendix B Table 2: Counts and percentage frequency for each indication for rHR from 2006-2019 combined

Indication for revision	n	Percent
Infection	10938	9.6
Malalignment/Size mismatch	1433	1.3
Adverse soft tissue reaction	5940	5.2
Loosening/Lysis	31796	27.8
Component Wear/Breakage	10625	9.3
Dislocation/Instability	16830	14.7
Fracture	11507	10.1
Unexplained pain	17286	15.1
Other	7993	7.0

Appendix C – Data Cleaning

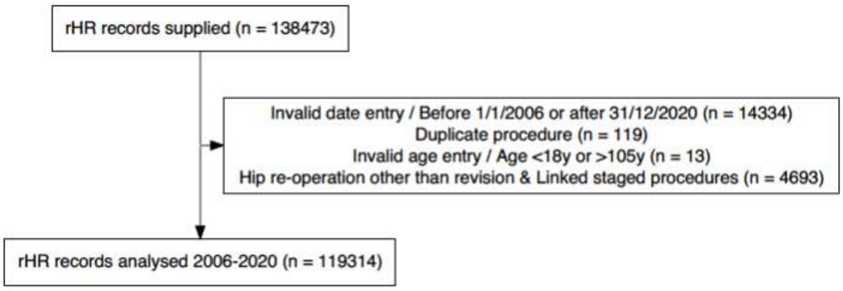

Appendix C Figure 1: Flowchart demonstrating attrition of study records during data cleaning.

Appendix D – Further analyses of trends over time

	2006	2007	2008	2009	2010	2011	2012	2013	2014	2015	2016	2017	2018	2019	2020
**Annual totals of rHR procedures**															
All rHR	4775	6322	6870	7274	7905	8869	10217	9562	9493	9134	8714	8709	8393	8111	4966
No linked primary	4570	5779	5915	5938	6098	6308	7001	6566	6346	5780	5334	5160	4624	4257	2408
First linked rHR	202	522	899	1234	1637	2309	2840	2634	2758	2896	2919	3044	3224	3297	2163
Second linked rHR	2	19	52	90	141	219	324	309	308	365	361	401	401	427	306
Third or more linked rHR	1	2	4	12	29	33	52	53	81	93	100	104	144	130	89
**Incidence rates of rHR**															
Crude incidence rate per 100,000 adults	11 (10.7 - 11.3)	14.4 (14 - 14.8)	15.5 (15.1 - 15.9)	16.3 (15.9 - 16.6)	17.5 (17.1 - 17.9)	19.5 (19.1 - 19.9)	22.3 (21.8 - 22.7)	20.7 (20.3 - 21.1)	20.4 (20 - 20.8)	19.4 (19 - 19.8)	18.4 (18 - 18.8)	18.3 (17.9 - 18.7)	17.5 (17.1 - 17.9)	16.8 (16.5 - 17.2)	10.2 (10 - 10.5)
Age-specific incidence rate per 100,000 persons															
18-49 years	1.3 (1.2 - 1.5)	1.5 (1.4 - 1.7)	1.8 (1.6 - 2)	2 (1.8 - 2.2)	2 (1.9 - 2.2)	2.7 (2.5 - 2.9)	2.9 (2.7 - 3.1)	2.2 (2 - 2.4)	2.1 (1.9 - 2.2)	2.1 (1.9 - 2.2)	1.9 (1.7 - 2)	1.6 (1.5 - 1.8)	1.4 (1.3 - 1.6)	1.3 (1.1 - 1.4)	0.9 (0.8 - 1)
50-59 years	8.5 (7.8 - 9.2)	11.2 (10.5 - 12.1)	11.3 (10.5 - 12.1)	12.1 (11.3 - 12.9)	14.6 (13.7 - 15.6)	17.4 (16.4 - 18.4)	19 (18 - 20.1)	16.3 (15.4 - 17.3)	14.6 (13.7 - 15.5)	13.3 (12.5 - 14.2)	12 (11.2 - 12.8)	11.5 (10.7 - 12.2)	10.2 (9.5 - 10.9)	10 (9.4 - 10.7)	6.2 (5.7 - 6.8)
60-69 years	23.8 (22.5 - 25.1)	30.2 (28.7 - 31.7)	32.5 (31.1 - 34.1)	33.2 (31.7 - 34.7)	34.6 (33.2 - 36.2)	38.6 (37 - 40.2)	44.9 (43.2 - 46.6)	40.8 (39.2 - 42.4)	37.7 (36.2 - 39.2)	35.2 (33.8 - 36.7)	32.2 (30.8 - 33.6)	30.3 (28.9 - 31.6)	28.4 (27.1 - 29.7)	27.3 (26 - 28.6)	14.1 (13.2 - 15.1)
70-79 years	45.9 (43.8 - 48.2)	61.2 (58.8 - 63.8)	63.6 (61.1 - 66.2)	67.6 (65 - 70.2)	70.9 (68.3 - 73.6)	75.3 (72.6 - 78)	87.5 (84.6 - 90.4)	81.5 (78.7 - 84.3)	79.8 (77.1 - 82.6)	74.2 (71.6 - 76.8)	71 (68.5 - 73.6)	70.4 (68 - 72.9)	67.6 (65.2 - 70)	60.8 (58.6 - 63)	35 (33.4 - 36.7)
80+ years	40.7 (38.1 - 43.5)	56.1 (53 - 59.3)	62.3 (59.1 - 65.7)	62.1 (58.9 - 65.4)	66.8 (63.5 - 70.2)	69.8 (66.5 - 73.3)	77.7 (74.2 - 81.3)	80.9 (77.4 - 84.6)	87.9 (84.2 - 91.6)	86.5 (82.9 - 90.2)	83 (79.5 - 86.6)	87.1 (83.6 - 90.8)	82.6 (79.2 - 86.2)	82.1 (78.7 - 85.6)	56.2 (53.4 - 59.1)

Appendix D Table 1: Annual totals and incidences rates of rHR

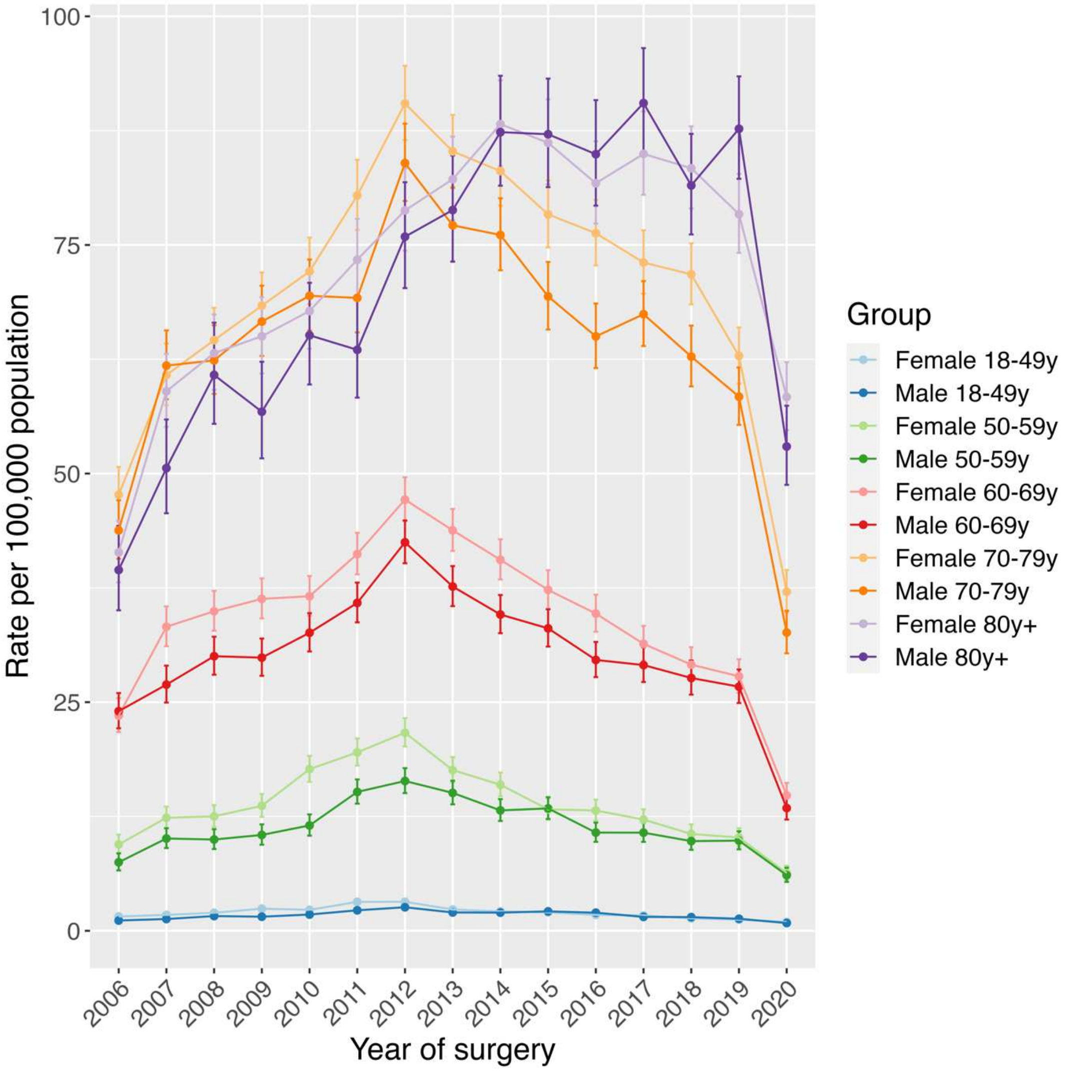

Appendix D Figure 1: Age- and gender-specific annual incidence rates for all rHR from 2006-2020. The rate of intervention was generally higher in female patients, though differences were smaller in more recent years.

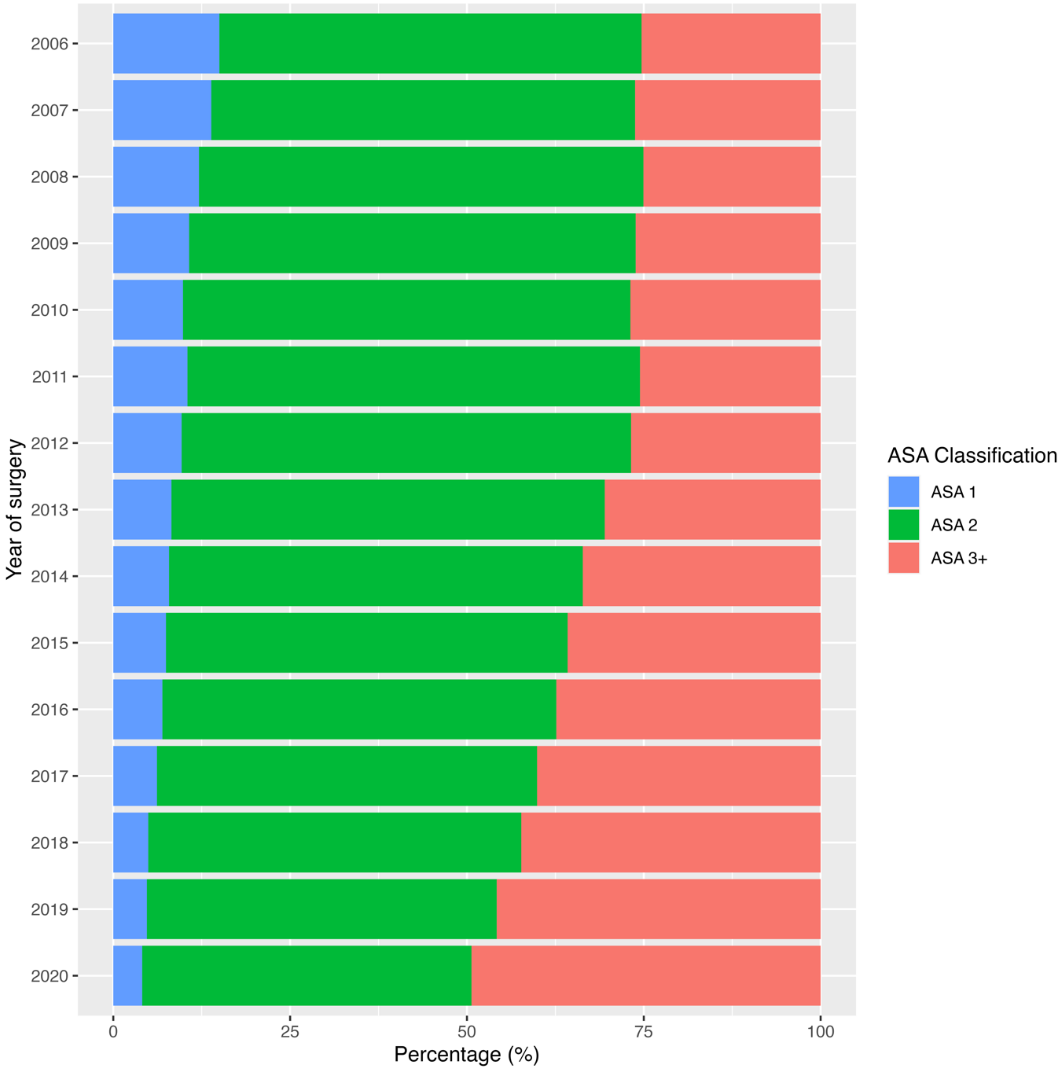

Appendix D Figure 2: Trends over time in the annual proportion of each American Society of Anesthesiologists (ASA) classification in patients undergoing all rHR between 2006-2020. The proportion of ASA Class 3+ patients increased from 25.3% in 2006 to 45.8% in 2019, with a further increase in 2020 (49.4%). Both ASA Class 1 and ASA Class 2 groups became smaller over the study period.

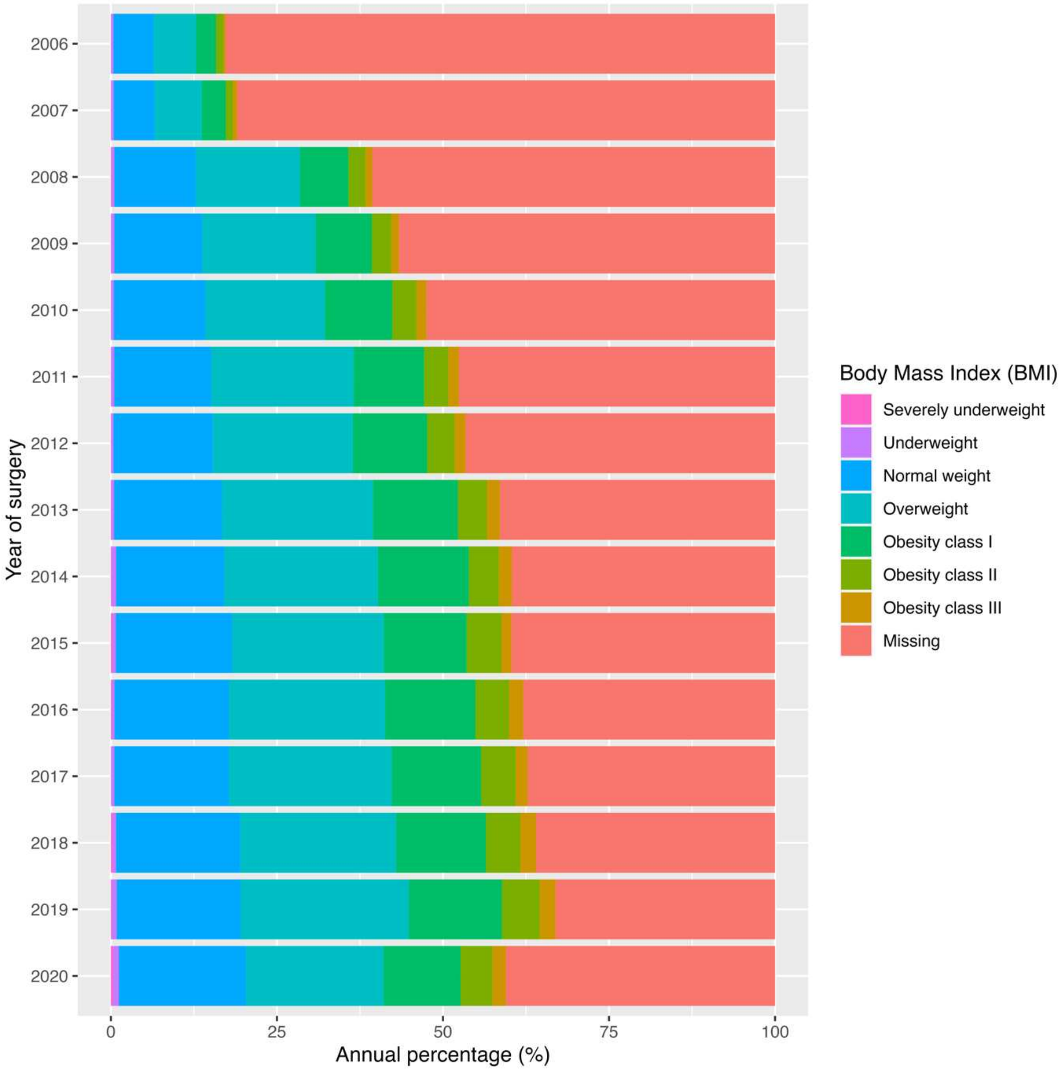

Appendix D Figure 3: Trends over time in the annual proportion of each Body Mass Index (BMI) classification in patients undergoing all rHR between 2006-2020. BMI was categorized following World Health Organisation cut-offs [4]. This figure demonstrates that BMI data were missing for a large proportion of procedures, with data collection improving over time. In 2006, 82.7% of records were missing BMI data, compared to 33.1% in 2019.

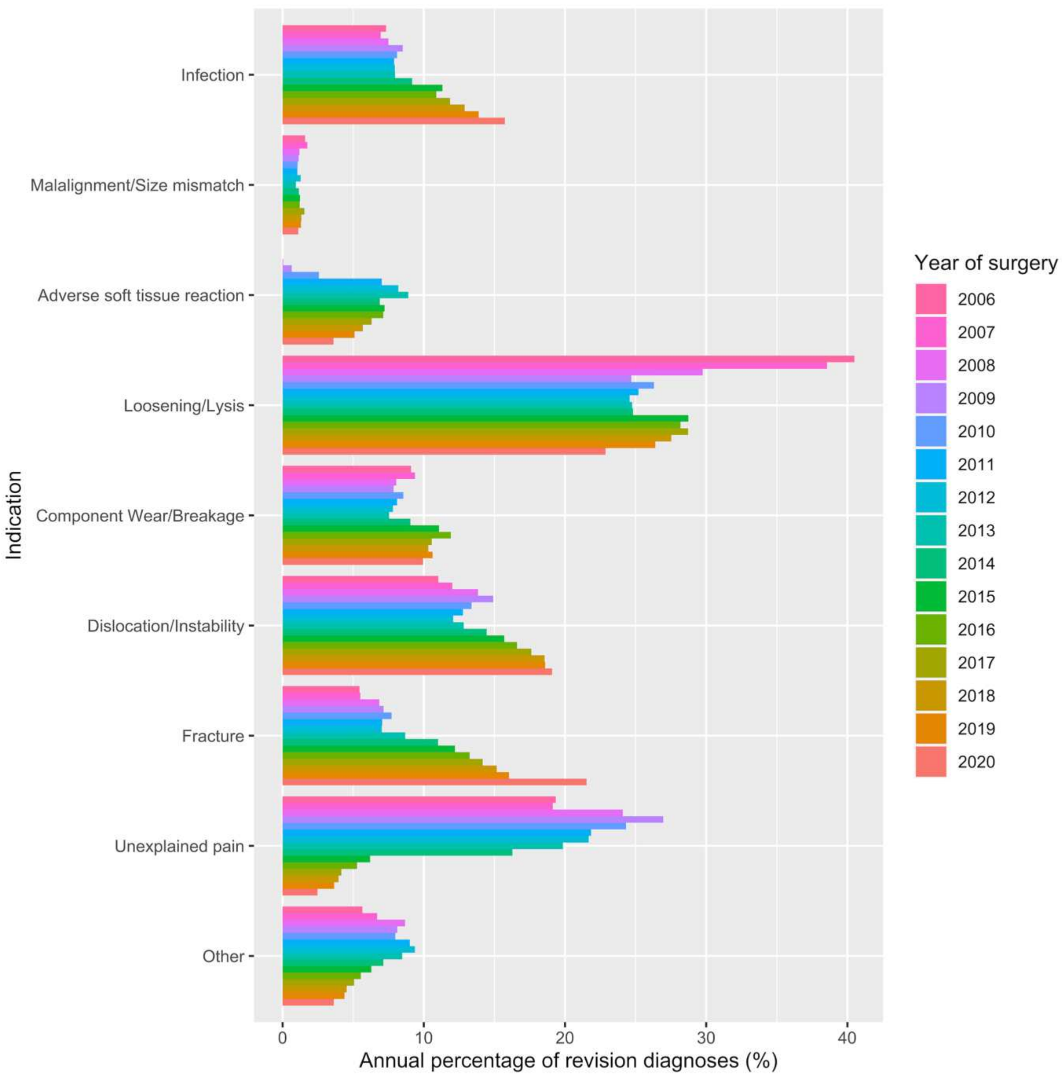

Appendix D Figure 4: Grouped barplots demonstrating changes in the annual proportions of each revision indication for all rHR from 2006-2020. Indications are ranked in hierarchical order (greatest importance at the top).

References

1. Sabah SA, Knight R, Alvand A, et al. No exponential rise in revision knee replacement surgery over the past 15 years: An analysis from the National Joint Registry. *Osteoarthritis and Cartilage*. Published online 2022. doi:10.1016/j.joca.2022.08.016

2. Office for National Statistics (ONS). Population estimates. Accessed November 19, 2021. https://www.ons.gov.uk/peoplepopulationandcommunity/populationandmigration/populationestimates

3. Public Health England. Technical guide: Confidence Intervals. Published online May 2018. Accessed November 19, 2021. https://fingertips.phe.org.uk/documents/PHDS%20Guidance%20-%20Confidence%20Intervals.pdf

4. Obesity: preventing and managing the global epidemic. Report of a WHO consultation. *World Health Organ Tech Rep Ser*. 2000;894:i-xii, 1-253.
