# Supplementary material for: Epidemiology of revision hip replacement surgery in the UK over the past 15 years—an analysis from the National Joint Registry
Source: BMJ Open. 2023 Oct 17;13(10):e072462. doi: 10.1136/bmjopen-2023-072462 (PMC10583040; doi:10.1136/bmjopen-2023-072462)
Supplement: Author's manuscript [file bmjopen-2023-072462.draft_revisions.pdf]

BMJ Open

BMJ Open is committed to open peer review. As part of this commitment we make the peer review history of every article we publish publicly available.

When an article is published we post the peer reviewers' comments and the authors' responses online. We also post the versions of the paper that were used during peer review. These are the versions that the peer review comments apply to.

The versions of the paper that follow are the versions that were submitted during the peer review process. They are not the versions of record or the final published versions. They should not be cited or distributed as the published version of this manuscript.

BMJ Open is an open access journal and the full, final, typeset and author-corrected version of record of the manuscript is available on our site with no access controls, subscription charges or pay-per-view fees (http://bmjopen.bmj.com).

If you have any questions on BMJ Open's open peer review process please email info.bmjopen@bmj.com

BMJ Open

Epidemiology of revision hip replacement surgery in the UK over the past 15 years - An analysis from the National Joint Registry

Journal:	*BMJ Open*
Manuscript ID	bmjopen-2023-072462
Article Type:	Original research
Date Submitted by the Author:	04-Feb-2023
Complete List of Authors:	Sabah, Shiraz; University of Oxford Nuffield Department of Orthopaedics Rheumatology and Musculoskeletal Sciences, Price Knee Research Group<br>Knight, Ruth; University of Oxford,<br>Nicolson, Philippa; University of Oxford, Nuffield Department of Orthopaedics, Rheumatology and Musculoskeletal Sciences<br>Taylor, Adrian; University of Oxford Nuffield Department of Orthopaedics Rheumatology and Musculoskeletal Sciences<br>Kendrick, Benjamin; University of Oxford Nuffield Department of Orthopaedics Rheumatology and Musculoskeletal Sciences<br>Alvand, Abtin; University of Oxford Nuffield Department of Orthopaedics Rheumatology and Musculoskeletal Sciences,<br>Petrou, Stavros; Nuffield Department of Primary Care Health Sciences,<br>Beard, David; University of Oxford, Nuffield Dept of Orthopaedics, Rheumatology and Musculoskeletal Sciences<br>Price, Andrew; University of Oxford, Nuffield Department of Orthopaedics, Rheumatology and Musculoskeletal Sciences<br>Palmer, Antony; University of Oxford, Nuffield Department of Orthopaedics, Rheumatology and Musculoskeletal Sciences
Keywords:	Hip < ORTHOPAEDIC & TRAUMA SURGERY, SURGERY, Orthopaedic & trauma surgery < SURGERY

SCHOLARONE™
Manuscripts

I, the Submitting Author has the right to grant and does grant on behalf of all authors of the Work (as defined in the below author licence), an exclusive licence and/or a non-exclusive licence for contributions from authors who are: i) UK Crown employees; ii) where BMJ has agreed a CC-BY licence shall apply, and/or iii) in accordance with the terms applicable for US Federal Government officers or employees acting as part of their official duties; on a worldwide, perpetual, irrevocable, royalty-free basis to BMJ Publishing Group Ltd ("BMJ") its licensees and where the relevant Journal is co-owned by BMJ to the co-owners of the Journal, to publish the Work in this journal and any other BMJ products and to exploit all rights, as set out in our licence.

The Submitting Author accepts and understands that any supply made under these terms is made by BMJ to the Submitting Author unless you are acting as an employee on behalf of your employer or a postgraduate student of an affiliated institution which is paying any applicable article publishing charge ("APC") for Open Access articles. Where the Submitting Author wishes to make the Work available on an Open Access basis (and intends to pay the relevant APC), the terms of reuse of such Open Access shall be governed by a Creative Commons licence – details of these licences and which Creative Commons licence will apply to this Work are set out in our licence referred to above.

Other than as permitted in any relevant BMJ Author's Self Archiving Policies, I confirm this Work has not been accepted for publication elsewhere, is not being considered for publication elsewhere and does not duplicate material already published. I confirm all authors consent to publication of this Work and authorise the granting of this licence.

For peer review only

Epidemiology of revision hip replacement surgery in the UK over the past 15 years - An analysis from the National Joint Registry

Authors

1. Shiraz A. Sabah, 0000-0003-2401-1372 – shiraz.sabah@ndorms.ox.ac.uk*
2. Ruth Knight, 0000-0001-6810-2845 – ruth.knight@liverpool.ac.uk
3. Philippa Nicolson, 0000-0003-2394-4867 – philippa.nicolson@ndorms.ox.ac.uk
4. Adrian Taylor – adrian.taylor@ndorms.ox.ac.uk
5. Benjamin Kendrick – ben.kendrick@ndorms.ox.ac.uk
6. Abtin Alvand, 0000-0001-6069-978X – abtin.alvand@ndorms.ox.ac.uk
7. Stavros Petrou, 0000-0003-3121-6050 - stavros.petrou@phc.ox.ac.uk
8. David J. Beard, 0000-0001-7884-6389 – david.beard@ndorms.ox.ac.uk
9. Andrew J. Price, 0000-0002-4258-5866 – andrew.price@ndorms.ox.ac.uk
10. Antony J. R. Palmer, 0000-0003-4616-7482 – antony.palmer@ndorms.ox.ac.uk

Institutions

1. Nuffield Department of Orthopaedics, Rheumatology and Musculoskeletal Sciences, University of Oxford

2. Nuffield Orthopaedic Centre, Oxford

For peer review only

3. Liverpool Clinical Trials Centre, University of Liverpool

4. Nuffield Department of Primary Care Health Sciences, Oxford

Initials, surnames, appointments, and highest academic degree

SA Sabah: DPhil Student, BSc FRCS(Orth) 1,2*

R Knight: Senior Medical Statistician, MMath, PhD 3

P Nicolson: Post-Doctoral Researcher, BPhty, PhD 1,2

A Taylor: Consultant Orthopaedic Surgeon, FRCS(Orth) 1,2

B Kendrick: Consultant Orthopaedic Surgeon, DPhil FRCS(Orth) 1,2

A Alvand: Consultant Orthopaedic Surgeon, DPhil FRCS(Orth) 1,2

S Petrou: Professor of Health Economics, BSc MPhil PhD 4

DJ Beard: Professor of Musculoskeletal and Surgical Science, DPhil, FRCS (hon) 1

AJ Price: Professor of Orthopaedics, DPhil FRCS(Orth) 1,2

AJR Palmer: Consultant Orthopaedic Surgeon, DPhil FRCS(Orth) 1,2

Correspondence address

*Corresponding author

Price Knee Research Group, Nuffield Department of Orthopaedics, Rheumatology and Musculoskeletal Sciences (NDORMS)

Botnar Research Centre, Oxford, OX3 7LD

For peer review only

Abstract

Objectives: To investigate trends in the incidence rate and main indication for revision hip replacement (rHR) over the past 15 years in the UK.

Design: Repeated national cross-sectional study from 2006 to 2020.

Setting/Participants: rHR procedures were identified from the National Joint Registry for England, Wales, Northern Ireland, the Isle of Man and the States of Guernsey. Population statistics were obtained from the Office for National Statistics.

Main outcome measures: Crude incidence rates of rHR.

Results: The incidence rate of rHR doubled from 11 per 100,000 adults in 2006 (95% CI 10.7 to 11.3) to a peak of 22 per 100,000 adults (95% CI 22 to 23) in 2012, before falling to 17 per 100,000 adults in 2019 (95% CI 16 to 17) [24.5% decrease from peak]. The incidence rate of rHR reduced by 39% in 2020 compared with 2019 (during the Covid-19 pandemic). The most frequent indications for rHR between 2006-2019 were loosening/lysis (27.8%), unexplained pain (15.1%) and dislocation/instability (14.7%). There were incremental increases in the annual number and incidence rates of rHR for fracture, infection, dislocation/instability, and a decrease for aseptic loosening/lysis.

For peer review only

Conclusions: The incidence rate of rHR doubled from 2006 to 2012, likely due to high early failure rates of metal-on-metal hip replacements. The incidence of rHR then decreased by approximately 25% from 2012 to 2019, followed by a profound decrease during the Covid-19 pandemic. The decrease in the number of rHR performed for aseptic loosening/lysis may reflect improved wear and implant longevity. Increased healthcare resource will be required to care for increased numbers of patients undergoing rHR for fracture or infection.

For peer review only

Article Summary

Strengths and limitations of this study:

- This study investigated national trends in rHR over a 15-year period.
- Surgeon-coded diagnoses were used to investigate trends in the indication for rHR.
- Crude incidence rates of rHR were calculated using arthroplasty records from the National Joint Registry and population statistics from the Office for National Statistics.
- Directly standardized rates of rHR were calculated based on the 2013 European Standard Population and may facilitate international comparison of arthroplasty practices.

Word count: 3264 words

Keywords: total hip replacement; revision; re-operation; epidemiology

For peer review only

Introduction

Primary hip replacement (pHR) is a highly successful intervention and is associated with large improvements in joint function and health-related quality of life (HRQoL) [1]. Prior to the Covid-19 pandemic, most international joint replacement registries and insurance databases reported increasing counts of pHR each year [2–4]. Nearly all analyses project an increase in the requirement for pHR over the coming decades [5–7], due to ageing populations and associated increases in the prevalence of hip osteoarthritis [8, 9].

Revision hip replacement (rHR) refers to a heterogeneous group of surgical procedures that usually involve the exchange or modification of one or more components of an existing hip replacement. It is performed infrequently compared with pHR and, following a pHR most patients do not require further surgery to that hip during their lifetime [10, 11]. However, increases in the number of pHR performed each year, and in younger patients, coupled with greater life expectancy, results in an increase in the number of pHR that are at risk of failure. This is expected to create a large increase in the number of rHR procedures required in the future [5]. The epidemiology and trends in rHR are not well understood.

The National Joint Registry (NJR) for England, Wales, Northern Ireland, the Isle of Man and the States of Guernsey provides information on annual total counts of rHR [12]. This registry data has shown that the total number of rHR in the UK peaked in 2012 and has since declined. This observation is thought to be due to the high utilisation of metal-on-metal (MOM) hip implants in the first decade of the 21st century, some of which had very high, early failure rates [12]. Count

For peer review only

data provide only a limited perspective. They are useful for understanding the number of surgeons and surgical units performing rHR, which may, for example, inform budget estimates. However, they do not provide information on the rate of intervention, or trends in different procedures and patient groups, which requires knowledge of the size and demographics of the general population. Count data are also of limited value for comparing UK practice with other countries. It is also important to understand whether the indications for rHR have temporally changed. In revision knee replacement (rKR), the number and proportion of procedures performed for infection have increased, whilst procedures for aseptic loosening have decreased [13, 14]. It is currently unknown whether a similar trend exists for rHR. The aim of our study was to investigate trends in the incidence rHR by indication during the past fifteen years in the UK, including the impact of the COVID-19 pandemic.

For peer review only

Patients and Methods

This study was reported according to the RECORD checklist [22].

Study dataset

The NJR prospectively collects data on pHR and rHR from hospital providers within its geographic remit. Data submission has been mandatory for independent sector providers since 2003 and for NHS providers since April 2011 [15]. rHR procedures are submitted to the NJR using H2 Minimum Data Set forms, which include fields on patient, surgeon, operation and implant characteristics. The current NJR definition of revision is "any operation where one or more components are added to, removed from or modified in a joint replacement or if a Debridement And Implant Retention (DAIR) with or without modular exchange is performed". The main change to this definition over the study period was the inclusion of DAIR procedures without modular exchange from 25/06/2018. Further information on all amendments to this definition can be found in the glossaries of NJR annual reports and the publication Operations included in the NJR [16].

Timeframe

This study examined NJR records for rHR performed between 1st January 2006 and 31st December 2020. We chose to exclude NJR records from 2003 to 2005, due to the known poor compliance with the registry when first formed [17, 18]. Records from 2021 were excluded because: (i) the NJR data extract was produced on 3rd February 2022, which meant a limited

For peer review only

window for late submission of data; and (ii) population statistics from the Office for National Statistics (ONS) were unavailable. We analysed longer-term trends using records from 2006 to 2019. We hypothesised that rHR activity in 2020 was unlikely to be representative of usual practice due to the disruption in arthroplasty services as a result of the Covid-19 pandemic. To measure the impact of the Covid-19 pandemic, we compared data from 2019 (pre-pandemic) to 2020 (pandemic).

Statistics

All statistical code is available from the lead author's GitHub page.

Trends over time in the incidence rate of rHR

Crude incidence rates of pHR and rHR were calculated for each calendar year from 2006 to 2020. The crude incidence rate was defined as the annual total count of procedures in a calendar year divided by the sum of the mid-year population estimates for adults in England, Wales and Northern Ireland. The *phe_rate* function within the PHEindicatormethods package in R was used to calculate 95% confidence intervals following Byar's method [19, 20]. In some situations, a rHR may be performed as a staged procedure with two distinct operations on different days. We considered first- and second-stage rHR as a single procedure to calculate incidence rates. The following criteria were applied to bundle these procedures: (i) The NJR H2 form completed at the first-stage procedure was required to specify the intention to treat the patient in stages, and (ii) the

For peer review only

second-stage 86 procedure was required to have been performed within 365 days of the first-stage. Analysis of hospital resource utilisation should count each stage individually, however, these totals are readily available in NJR annual reports we did not reproduce this analysis [2]. The crude incidence rate was presented as a line graph overlying bars of annual total counts. Long term trends in total counts and incidence rates were analysed descriptively from 2006 to 2019. The percentage decrease in the total count of rHR during the Covid pandemic was analysed as the change in total count from 2019 to 2020, divided by the total count in 2019. The percentage decrease in the incidence rate of rHR was calculated following the same principles.

We investigated trends over time in the number of rHR procedures linked to a pHR on the NJR. This analysis is presented as stacked bars of annual total counts for linked and not linked procedures. Our dataset did not allow us to compare incidence rates for first and subsequent re-revision procedures because the proportion of linked procedures changed markedly over the study period. For that reason, we were also unable to make a meaningful comparison between first rHR of metal-on-metal pHR compared with other types of pHR. The incidence rate of rHR within a given year is not strongly associated with the incidence rate of pHR in that year, because few pHR fail within one year [14]. The prevalence of pHR and rHR in the general population is more relevant, however, we were unable to estimate prevalence accurately given the longevity of hip replacements [11] and the comparatively short existence of the NJR. To enable future comparisons to populations with different age-structures (for example, from different countries around the world) we calculated directly standardised rates (DSRs) for pHR and rHR. This methodology is described in Appendix B and uses the 2013 European Standard Population.

For peer review only

Trends over time in the incidence rate of rHR by patient age

The study population was stratified into the following groups to investigate trends over time in the incidence rate of rHR by age at the time of revision surgery: 18-49 years/ 50-59 years/ 60-69 years/ 70-79 years/ 80+ years. Age-specific incidence rates per 100,000 persons were calculated for each group for each year of analysis and presented graphically.

Trends in the main indication for rHR

Each rHR procedure was assigned a single, dominant indication for surgery using a diagnosis hierarchy based on the Australian Orthopaedic Association National Joint Replacement Registry (AOANJRR) model [21] (Appendix C). Pre-pandemic data (2006 to 2019) were combined to calculate the percentage frequency of each indication. To investigate trends over time, crude incidence rates per 100,000 adults and annual percentage frequencies were calculated for each indication in each year (from 2006 to 2020, i.e. including pandemic data). These data were presented using grouped barplots.

To investigate the proportional share of each revision indication in first- and subsequent re-revision rHR, we analysed data from the five years immediately pre-pandemic (2014 to 2019). We excluded earlier NJR data as we considered it to be most vulnerable to the maturity of the registry. We defined the following groups: first linked rHR (the earliest revision procedure for a given patient-side linked to a primary hip replacement on the NJR); second linked rHR (the next revision

For peer review only

procedure for a given patient-side linked to a first linked rHR); third or more linked rHR (subsequent revision procedure(s) linked to a second linked rHR); and no linked primary (revision procedure(s) not linked to a primary procedure). These data were presented using barplots.

Software

Statistical analyses were performed using R version 4.2.1.

Patient and Public Involvement

None.

For peer review only

Results

Data cleaning and linkage

The attrition of study records during data cleaning is illustrated in the Supplementary Material Appendix A Figure 1.

Trends over time in the incidence rate of rHR

Annual total counts and crude incidence rates of rHR procedures reported to the NJR are presented in Table 1 and Figure 1. Annual total counts increased every year from 2006 (4775 rHR) to 2012 (10,217 rHR) [114% increase]. Following this peak, annual total counts declined, with 8111 rHR performed in 2019 [20.6% decrease from peak]. The incidence rate of rHR increased from 11.0 per 100,000 adults in 2006 (95% CI 10.7 to 11.3) to 22.3 per 100,000 adults in 2012 (95% CI 21.8 to 22.7), before falling to 16.8 per 100,000 adults in 2019 (95% CI 16.5 to 17.2) [24.5% decrease from peak]. In 2020, where rHR practice was affected by the Covid-19 pandemic, 4966 rHR procedures were reported to the NJR: a crude incidence rate of 10.2 per 100,000 adults (95% CI 10.0 to 10.5). These figures represented a 38.8% reduction in the total count of rHR and a 39.0% reduction in the incidence rate of rHR in 2020 compared to 2019. The percentage of rHR procedures linked to a pHR on the NJR increased from 4.3% in 2006 to 47.5% in 2019 (rising further to 51.5% in 2020) (Figure 2). The annual total counts of pHR increased every year from 2006 (48,700 pHR) to 2019 (101,828 pHR). However, increases were smaller during the last four years. The incidence rate of pHR increased from 112 per 100,000 adults in 2006 (95% CI 111 to 113) to 211 per 100,000 adults in 2019 (95% CI 210 to 212). This represented a 109.1% increase in the total count and 88.6% increase in the incidence rate of pHR from 2006 to 2019. In 2020,

For peer review only

240 during the Covid-19 pandemic, 57,437 pHR procedures were reported to the NJR: a crude
241 incidence rate of 119 per 100,000 adults (95% CI 118 to 120). This represented a 43.6% reduction
242 in the total count of pHR and a 43.8% reduction in the incidence rate of pHR in 2020 compared to
243 2019.
244
245

For peer review only

Table 1: Annual totals and incidences rates of rHR

	2006	2007	2008	2009	2010	2011	2012	2013	2014	2015	2016	2017	2018	2019	2020
**Annual totals of rHR procedures**															
All rHR	4775	6322	6870	7274	7905	8869	10217	9562	9493	9134	8714	8709	8393	8111	4966
No linked primary	4570	5779	5915	5938	6098	6308	7001	6566	6346	5780	5334	5160	4624	4257	2408
First linked rHR	202	522	899	1234	1637	2309	2840	2634	2758	2896	2919	3044	3224	3297	2163
Second linked rHR	2	19	52	90	141	219	324	309	308	365	361	401	401	427	306
Third or more linked rHR	1	2	4	12	29	33	52	53	81	93	100	104	144	130	89
**Incidence rates of rHR**															
Crude incidence rate per 100,000 adults	11 (10.7 - 11.3)	14.4 (14 - 14.8)	15.5 (15.1 - 15.9)	16.3 (15.9 - 16.6)	17.5 (17.1 - 17.9)	19.5 (19.1 - 19.9)	22.3 (21.8 - 22.7)	20.7 (20.3 - 21.1)	20.4 (20 - 20.8)	19.4 (19 - 19.8)	18.4 (18 - 18.8)	18.3 (17.9 - 18.7)	17.5 (17.1 - 17.9)	16.8 (16.5 - 17.2)	10.2 (10 - 10.5)
Age-specific incidence rate per 100,000 persons															
18-49 years	1.3 (1.2 - 1.5)	1.5 (1.4 - 1.7)	1.8 (1.6 - 2)	2 (1.8 - 2.2)	2 (1.9 - 2.2)	2.7 (2.5 - 2.9)	2.9 (2.7 - 3.1)	2.2 (2 - 2.4)	2.1 (1.9 - 2.2)	2.1 (1.9 - 2.2)	1.9 (1.7 - 2)	1.6 (1.5 - 1.8)	1.4 (1.3 - 1.6)	1.3 (1.1 - 1.4)	0.9 (0.8 - 1)
50-59 years	8.5 (7.8 - 9.2)	11.2 (10.5 - 12.1)	11.3 (10.5 - 12.1)	12.1 (11.3 - 12.9)	14.6 (13.7 - 15.6)	17.4 (16.4 - 18.4)	19 (18 - 20.1)	16.3 (15.4 - 17.3)	14.6 (13.7 - 15.5)	13.3 (12.5 - 14.2)	12 (11.2 - 12.8)	11.5 (10.7 - 12.2)	10.2 (9.5 - 10.9)	10 (9.4 - 10.7)	6.2 (5.7 - 6.8)
60-69 years	23.8 (22.5 - 25.1)	30.2 (28.7 - 31.7)	32.5 (31.1 - 34.1)	33.2 (31.7 - 34.7)	34.6 (33.2 - 36.2)	38.6 (37 - 40.2)	44.9 (43.2 - 46.6)	40.8 (39.2 - 42.4)	37.7 (36.2 - 39.2)	35.2 (33.8 - 36.7)	32.2 (30.8 - 33.6)	30.3 (28.9 - 31.6)	28.4 (27.1 - 29.7)	27.3 (26 - 28.6)	14.1 (13.2 - 15.1)
70-79 years	45.9 (43.8 - 48.2)	61.2 (58.8 - 63.8)	63.6 (61.1 - 66.2)	67.6 (65 - 70.2)	70.9 (68.3 - 73.6)	75.3 (72.6 - 78)	87.5 (84.6 - 90.4)	81.5 (78.7 - 84.3)	79.8 (77.1 - 82.6)	74.2 (71.6 - 76.8)	71 (68.5 - 73.6)	70.4 (68 - 72.9)	67.6 (65.2 - 70)	60.8 (58.6 - 63)	35 (33.4 - 36.7)
80+ years	40.7 (38.1 - 43.5)	56.1 (53 - 59.3)	62.3 (59.1 - 65.7)	62.1 (58.9 - 65.4)	66.8 (63.5 - 70.2)	69.8 (66.5 - 73.3)	77.7 (74.2 - 81.3)	80.9 (77.4 - 84.6)	87.9 (84.2 - 91.6)	86.5 (82.9 - 90.2)	83 (79.5 - 86.6)	87.1 (83.6 - 90.8)	82.6 (79.2 - 86.2)	82.1 (78.7 - 85.6)	56.2 (53.4 - 59.1)

For peer review only - http://bmjopen.bmj.com/site/about/guidelines.xhtml

Trends over time in the incidence rate of rHR by patient age

The incidence rate of rHR increased in all age groups from 2006 to 2012 (Figure 3). The group with the largest increase in the incidence rate of rHR over this period was patients aged 70-79 years at the time of surgery, from 46 per 100,000 adults in 2006 [95% CI 44 to 48] to 87 per 100,000 adults in 2012 [95% CI 85 to 90]. The incidence rate of rHR declined for all age groups from 2012 to 2019, except patients aged 80 years or older at the time of surgery. In this patient group, the incidence rate of rHR peaked in 2014 (88 per 100,000 adults [95% CI 84 to 92]) and remained relatively unchanged through to 2019.

Trends in the main indication for rHR

During the years 2006 to 2019 combined, aseptic loosening was the most frequent indication for rHR (27.8%), followed by unexplained pain (15.1%) and then dislocation/instability (14.7%) (Appendix C). The incidence rate of rHR for infection increased nearly every year from 2006 (0.8 per 100,000 adults [95% CI 0.7 to 0.9]) to 2019 (2.3 per 100,000 adults [95% CI 2.2 to 2.5]) (Figure 4). A similar trend was observed for rHR for dislocation/instability and fracture. The incidence rate for malalignment/size mismatch changed little over the study period (~0.2 per 100,000 adults). For the remaining indications, incidence rates increased to a peak before decreasing in more recent years. rHR for adverse soft tissue reaction, unexplained pain, and 'other' peaked in 2012 and 2013. rHR for loosening/lysis and component wear/breakage peaked later, in 2015 and 2016 respectively.

For peer review only

Trends in the annual *proportional share* of each revision indication are presented in the supplementary material (Appendix D Figure 1). These data should be interpreted in conjunction with crude incidence rates. For example, whilst the proportion of rHR for loosening/lysis was lower in 2019 compared to 2006, the crude incidence rate was greater.

The proportion of revisions attributable to each indication within first-linked rHR and re-revision rHR from 2014-2019 is demonstrated in Figure 5. A linked pHR could not be found on the NJR for the majority of rHR performed over this period (40.1% [n= 21053/52554] linked). Among the rHR that could not be linked to a pHR, loosening/lysis was the most frequent diagnosis (33.3%, n= 10,500/31,501). Within first-linked rHR, loosening/lysis (19%, n= 3448/18138) and dislocation/instability (18.8%, n= 3409/18138)) were the most frequent diagnoses. Within linked re-revision rHR, infection was the most frequent diagnosis (31.1% second linked rHR, n= 703/2263; and 41.0% third or more linked rHR, n= 267/652).

For peer review only

Discussion

The total number and incidence rate of rHR doubled from 2006 to 2012, likely driven by high early failure rates of some types of metal-on-metal hip replacement (MOM-HR). The incidence rate of rHR then fell in subsequent years, and was three-quarters of its peak by 2019, suggesting improved pHR longevity. The total number and incidence rate of pHR reported to the NJR approximately doubled from 2006 to 2019, with increases every year until the Covid-19 pandemic. In 2020, during the Covid-19 pandemic, approximately 40% fewer rHR were performed compared with 2019. Patients aged 80 years and older displayed the largest increase in the rate of rHR during the study period. Contrary to younger age groups, where the count and rate of rHR procedures decreased after 2012, rHR activity in this older patient group was maintained. Between the years 2006 and 2019, aseptic loosening was the most frequent indication for rHR (27.8%), followed by unexplained pain (15.1%) and then dislocation/instability (14.7%). There were incremental increases in annual counts and incidence rates of rHR for infection, dislocation/instability and fracture over the study period, whereas rHR for loosening/lysis decreased in absolute and proportional terms.

Our findings differ from previous studies that forecast progressive increases in rHR activity [5, 23, 24]. The peak of rHR in 2012 is likely to reflect high early failure rates of MOM-HR prostheses, which were implanted in large numbers during the first decade of the 21st century. A significant proportion of primary MOM-HR predate the NJR. The increase in the incidence of rHR for adverse soft tissue reaction to particulate debris up until 2012/13 and subsequent decrease in this indication supports the conclusion. However, it is important to note that this indication was only added to the

For peer review only

H2 MDS form in 2008 and is not exclusive to MOM-HR. Loosening/lysis was the most frequent indication for rHR overall, and for each year studied except in 2009, when it was unexplained pain. We observed a decrease in the rate and proportion of rHR for loosening/lysis in the most recent years studied, which may represent low wear rates and improved longevity of modern implants. The increase in rate of rHR for instability is unexpected given cohort studies suggest there has been a fall in the incidence of dislocation, however, the threshold for revision following dislocation may have changed over time. Similar to other studies, we observed large increases in rHR for infection and fracture over the study period [24].

This study is the first to provide crude incidence rates, age-specific rates and directly standardised rates for rHR in the UK using NJR data. The strengths of this study include its reproducibility and openness. The study report was written in RMarkdown with all results derived from the source data, and statistical code made available to readers. However, the study does have limitations. Our study design is not able to differentiate between changes in reporting and changes in surgical practice. There is evidence that compliance with the NJR increased over the study period [2, 17, 18], which suggests that the true decrease in rHR in recent years may be greater than our estimates. The hierarchy we have used to assign a single, dominant indication for rHR may not reflect the true diagnosis in all cases. The coding of indications for surgery was modified over the study period (for example, unexplained pain was originally termed pain on data collection forms). The indications reported to the NJR reflect those thought to apply at the time of surgery. In the case of infection, which is sometimes diagnosed post-operatively, there is evidence to suggest it may be under-recognised by registries [25]. Procedures that are not considered a revision procedure - for example, operative fixation of a periprosthetic fracture, were not included in the study. There is

For peer review only

continued uncertainty whether periprosthetic hip fractures should be managed with revision or fixation, and trends will have changed with time [26, 27]. Our subgroup analysis of first-linked, second-linked and third or more-linked rHR excluded procedures with no pHR recorded in the registry. It is likely that our analyses over-represent failure modes occurring in early follow-up (such as infection or fracture) and under-represent failure modes more likely to occur at later timepoints (such as aseptic loosening/osteolysis and component wear). We attempted to mitigate this effect by restricting our analysis to more mature registry data (2014 to 2019), but longer-term data are required. We have not projected future requirements for rHR.

Our study demonstrates the considerable disruption to rHR practice during the Covid-19 pandemic, similar to trends recently reported for pHR, pKR and rKR [14, 28]. In the short-term, the number of rHR performed is likely to increase as a result of delayed care during the Covid-19 pandemic [29]. If current trends continue, fracture, infection and instability will represent the indication for an increasing proportion of rHR procedures, which has implications for healthcare provision. There are currently major infrastructure projects to increase the capacity for primary and revision joint replacement in the UK. These include development of surgical hubs to separate elective and emergency care, with the idea that planned surgical activity may continue with fewer disruptions during surges of unplanned hospital admissions [30].

In conclusion, the incidence rate of rHR doubled from 2006 to 2012, likely due to high early failure rates of metal-on-metal hip replacements. The incidence of rHR decreased by approximately 25% from 2012 to 2019, likely due to increased implant longevity. Recent trends suggest a rise in rHR

For peer review only

351 for fracture, infection, and dislocation/instability, and a fall in rHR for loosening/lysis. There was

352 a large reduction in the incidence of rHR during the Covid-19 pandemic in 2020.

353

354

For peer review only

Figure legends

Figure 1: Annual crude incidence rates (line, left y-axis) and total counts (bars, right y-axis) of (a) pHR and (b) rHR from 2006-2020. Note the differences in y-axis scales for pHR and rHR

Figure 2: Changes in the annual total counts of rHR that could be linked to a pHR on the NJR over time

Figure 3: Annual incidence rates of first linked rHR from 2006-2020 by age group.

Figure 4: Grouped barplots demonstrating annual incidence rates for all rHR by indication for surgery from 2006-2020. Diagnoses are ranked in hierarchical order (greatest importance at the top).

Figure 5: Percentage frequency of each indication for first and re-revision HR from 2014 to 2019. Diagnoses are ranked in hierarchical order (greatest importance at the top). For a given procedure (e.g. First linked rHR), the sum of all diagnoses is 100 percent.

For peer review only

Contributorship statement

All authors made substantial contributions to the work. CRediT roles:

- SAS: Conceptualisation, Data curation, Formal analysis, Funding acquisition, Investigation, Methodology, Software, Writing - original draft
- RK: Data curation, Formal analysis, Statistical expertise, Investigation, Methodology, Software, Writing - review & editing
- PN: Investigation, Methodology, Software, Writing - review & editing
- AT: Investigation, Writing - review & editing
- BK: Investigation, Writing - review & editing
- AA: Investigation, Writing - review & editing
- SP: Investigation, Writing - review & editing
- DJB: Conceptualisation, Funding acquisition, Investigation, Methodology, Supervision, Writing - review & editing
- AJP: Conceptualisation, Funding acquisition, Investigation, Methodology, Supervision, Writing - review & editing
- AJRP: Conceptualisation, Investigation, Methodology, Supervision, Writing - review & editing

For peer review only

Competing interests

SAS is funded by an NIHR Doctoral Research Fellowship, Royal College of Surgeons One Year Fellowship and funding from the Rosetrees Trust, and is a member of the editorial board of the Bone & Joint Journal. AA has received funding from Zimmer Biomet and Medacta International outside of the submitted work. AJP has received funding from Zimmer Biomet outside of the submitted work and is a board member of the British Orthopaedic Association and British Association for Surgery of the Knee. BK has received funding from Adler Ortho outside of the submitted work. SP is an NIHR Senior Investigator. DJB has received institutional funding from the NIHR and Rosetrees Trust.

Funding

This study received funding from the National Institute of Health Research (Doctoral Research Fellowship 301771), Royal College of Surgeons of England (Grant no. N/A) and Rosetrees Trust (Grant no. N/A. None of the funding sources had any role in the design or conduct of the study.

Data sharing statement

Data is available from the National Joint Registry following approval by their Research Subcommittee. Statistical code is available as a repository on the lead author's GitHub page.

Ethics approval statement

For peer review only

Ethical approval was obtained from the London-Bromley Research Ethics Committee (20/LO/0428). Data access approvals were obtained from the National Joint Registry (NJR) for England, Wales, Northern Ireland, the Isle of Man and the States of Guernsey (RSC2017/26). Data for patients who did not to consent to the NJR audit were not included.

For peer review only

Acknowledgements

We thank the patients and staff of all the hospitals who have contributed data to the National Joint Registry. We are grateful to the Healthcare Quality Improvement Partnership (HQIP), the NJR Research Committee and staff at the NJR for facilitating this work. The authors have conformed to the NJR's standard protocol for data access and publication. The views expressed represent those of the authors and do not necessarily reflect those of the National Joint Registry Steering Committee, Research Sub-committee or the Healthcare Quality Improvement Partnership (HQIP) who do not vouch for how the information is presented.

For peer review only

References

[1] S. A. Sabah, A. Alvand, D. J. Beard & A. J. Price, Minimal important changes and differences were estimated for Oxford hip and knee scores following primary and revision arthroplasty, Journal of Clinical Epidemiology 143 (2022) 159–168. doi:10.1016/j.jclinepi.2021.12.016. URL https://www.sciencedirect.com/science/article/pii/S0895435621004212

[2] National Joint Registry for England Wales Northern Ireland and the Isle of Man, NJR 18th Annual Report (September 2021) (2021). URL https://www.njrcentre.org.uk/njrcentre/Reports-Publications-and-Minutes/Annual-reports

[3] Australian Orthopaedic Association, Hip, Knee & Shoulder Arthroplasty Annual Report (2021). URL https://aoanjrr.sahmri.com/annual-reports-2021

[4] Swedish Arthroplasty Register, Annual Report 2021 (2021). URL https://registercentrum.blob.core.windows.net/slr/r/SAR-Annual-Report-2021-SJlAFmlRl5.pdf

[5] S. Kurtz, K. Ong, E. Lau, F. Mowat & M. Halpern, Projections of Primary and Revision Hip and Knee Arthroplasty in the United States from 2005 to 2030, The Journal of Bone and Joint Surgery (American) 89 (4) (2007) 780. doi:10.2106/JBJS.F.00222.

[6] J. A. Singh, S. Yu, L. Chen & J. D. Cleveland, Rates of Total Joint Replacement in the United States: Future Projections to 2020–2040 Using the National Inpatient Sample, The Journal of Rheumatology 46 (9) (2019) 1134–1140, publisher: The Journal of Rheumatology Section: Osteoarthritis 2019. doi:10.3899/jrheum.170990. URL https://www.jrheum.org/content/46/9/1134

[7] G. Matharu, D. Culliford, A. Blom & A. Judge, Projections for primary hip and knee replacement surgery up to the year 2060: An analysis based on data from The National Joint Registry for England, Wales, Northern Ireland and the Isle of Man, The Annals of The Royal College of Surgeons of EnglandPublisher: The Royal College of Surgeons of England (Dec. 2021). doi:10.1308/rcsann.2021.0206. URL https://publishing.rcseng.ac.uk/doi/full/10.1308/rcsann.2021.0206

[8] D. Spitaels et al., Epidemiology of knee osteoarthritis in general practice: A registry-based study, BMJ Open 10 (1) (2020) e031734, https://bmjopen.bmj.com/content/10/1/e031734

[9] M. McKee et al., The changing health needs of the UK population, The Lancet 397 (10288) (2021) 1979–1991, iSBN: 0140-6736 Publisher: Elsevier. doi:https://doi.org/10.1016/S0140-6736(21)00229-4.

[10] L. E. Bayliss et al., The effect of patient age at intervention on risk of implant revision after total replacement of the hip or knee: A population-based cohort study, The Lancet 389 (10077) (2017) 1424–1430. doi:10.1016/S0140-6736(17)30059-4.

[11] J. T. Evans et al., How long does a hip replacement last? A systematic review and meta-analysis of case series and national registry reports with more than 15 years of follow-up, Lancet 393 (10172) (2019) 647–654, URL http://dx.doi.org/10.1016/S0140-6736(18)32531-5

[12] National Joint Registry for England Wales Northern Ireland and the Isle of Man, NJR 19th Annual

Report (September 2022) (2022). URL https://reports.njrcentre.org.uk/downloads

[13] P. L. Lewis et al., Increases in the rates of primary and revision knee replacement are reducing: A 15-Year registry study across 3 continents, Acta Orthopaedica 91 (4) (2020) 414–419, URL https://doi.org/10.1080/17453674.2020.1749380

[14] S. A. Sabah et al., No exponential rise in revision knee replacement surgery over the past 15 years: An analysis from the National Joint Registry, Osteoarthritis and Cartilage doi: 10.1016/j.joca.2022.08.016.

[15] C. Boulton et al., Implementing large-scale data quality validation in a national arthroplasty registry to improve compliance, Bone & Joint Open 3 (9) (2022) 716–725 URL https://online.boneandjoint.org.uk/doi/full/10.1302/2633-1462.39.BJO-2022-367 0051.R1

[16] National Joint Registry for England Wales Northern Ireland, the Isle of Man and the States of Guernsey, Operations included in the NJR - Version 8, Tech. rep. (Jun. 2018). URL https://www.njrcentre.org.uk/wp-content/uploads/Operations-included-in-NJR371v8.pdf

[17] S. A. Sabah et al., Validation of primary metal-on-metal hip arthroplasties on the National Joint Registry for England, Wales and Northern Ireland using data from the London Implant Retrieval Centre: A study using the NJR dataset, The bone & joint journal 97-B (1) (2015) 10–18. doi:10.1302/0301-620X.97B1.35279.

[18] S. A. Sabah et al., Are all metal-on-metal hip revision operations contributing to the National Joint Registry implant survival curves?, Bone and Joint Journal 98B (1) (2016) 33–39. doi:10.1302/0301-620X.98B1.36431.

[19] Public Health England, Technical guide: Confidence Intervals (May 2018). URL https://fingertips.phe.org.uk/documents/PHDS%20Guidance%20-%20Confidence%20Intervals.pdf

[20] A. Georgina, F. Sebastian, F. Matthew, F. Paul & C. Emma, PHEindicatormethods: Common Public Health Statistics and their Confidence Intervals, (Jun. 2020). URL https://CRAN.R-project.org/package=PHEindicatormethods

[21] S. Graves, D. Davidson, R. de Steiger & A. Tomkins, Australian Orthopaedic Association National Joint Replacement Registry Annual Report 2010, Sydney, Australia: Australian Medical Association (2009) 1–200, iSBN: 9789197937849.

[22] E. I. Benchimol et al., The REporting of studies Conducted using Observational Routinely-collected health Data (RECORD) Statement, PLOS Medicine 12 (10) (2015) e1001885,. doi:10.1371/journal.pmed.1001885.

[23] A. Patel, G. Pavlou, R. E. Mújica-Mota & A. D. Toms, The epidemiology of revision total knee and hip arthroplasty in England and Wales: A comparative analysis with projections for the United States. a study using the national joint registry dataset, Bone and Joint Journal 97-B (8) (2015) 1076–1081, doi:10.1302/0301-620X.97B8.35170.

[24] A. M. Schwartz, K. X. Farley, G. N. Guild & T. L. Bradbury, Projections and Epidemiology of Revision Hip and Knee Arthroplasty in the United States to 2030, The Journal of Arthroplasty 35 (6) (2020) S79–S85, publisher: doi:10.1016/j.arth.2020.02.030.

For peer review only

482 [25] Z. P. Sinagra et al., The accuracy of reporting of periprosthetic joint infection to the Australian Orthopaedic Association National Joint
483 Replacement Registry, Bone & Joint Open 3 (5) (2022) 367–374, doi:10.1302/2633-1462.35.BJO-2022-0011.R1.

484 [26] J. L. Maggs et al., B2 or not B2? That is the question: a review of periprosthetic fractures around cemented taper-slip femoral components,
485 The Bone & Joint Journal 103-B (1) (2021) 71–78, doi:10.1302/0301-620X.103B1.BJJ-2020-0163.R1.

486 [27] M. F. R. Powell-Bowns et al., Vancouver B periprosthetic fractures involving the Exeter cemented stem, The Bone & Joint Journal 103-B (2)
487 (2021) 309–320, doi:10.1302/0301-620X.103B2.BJJ-2020-0695.R1.

488 [28] A. E. Sayers et al., The COVID-19 induced joint replacement deficit in England, Wales and Northern Ireland, National Joint Registry 18th
489 Annual Report. URL https://www.ncbi.nlm.nih.gov/books/NBK576854/

490 [29] Royal College of Surgeons of England, Surgeons call for investment in surgical hubs as NHS waiting list hits record 5.3 million (2021). URL
491 https://www.rcseng.ac.uk/news-and-events/media-centre/press-releases/waiting-times-may-2021/

492 [30] Royal College of Surgeons of England, The case for surgical hubs (2022). URL https://www.rcseng.ac.uk/about-the-rcs/government-relations-
493 and-consultation/position-statements-and-reports/the-case-for-surgical-hubs/

494 [31] Office for National Statistics (ONS), Population estimates. URL
495 https://www.ons.gov.uk/peoplepopulationandcommunity/populationandmigration/populationestimates

For peer review only

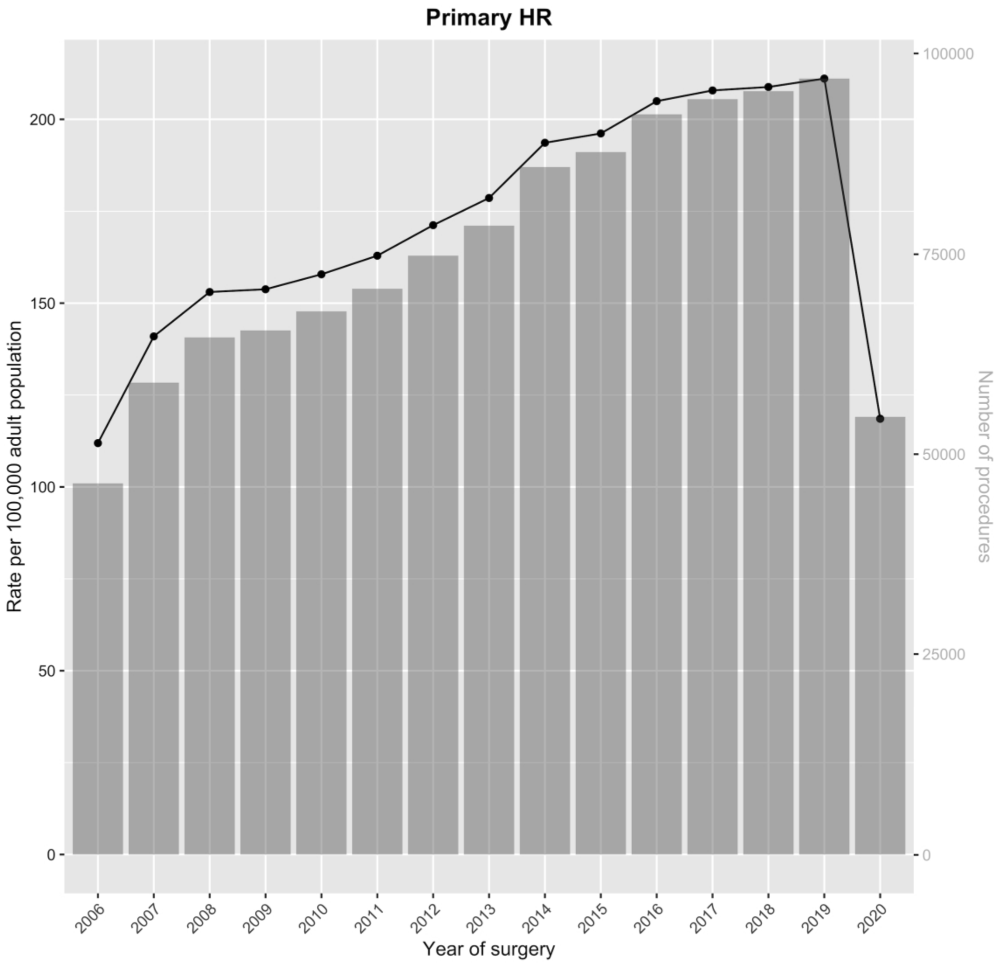

Figure 1a

Figure 1: Annual crude incidence rates (line, left y-axis) and total counts (bars, right y-axis) of (a) pHR and (b) rHR from 2006-2020. Note the differences in y-axis scales for pHR and rHR

1000x1000mm (72 x 72 DPI)

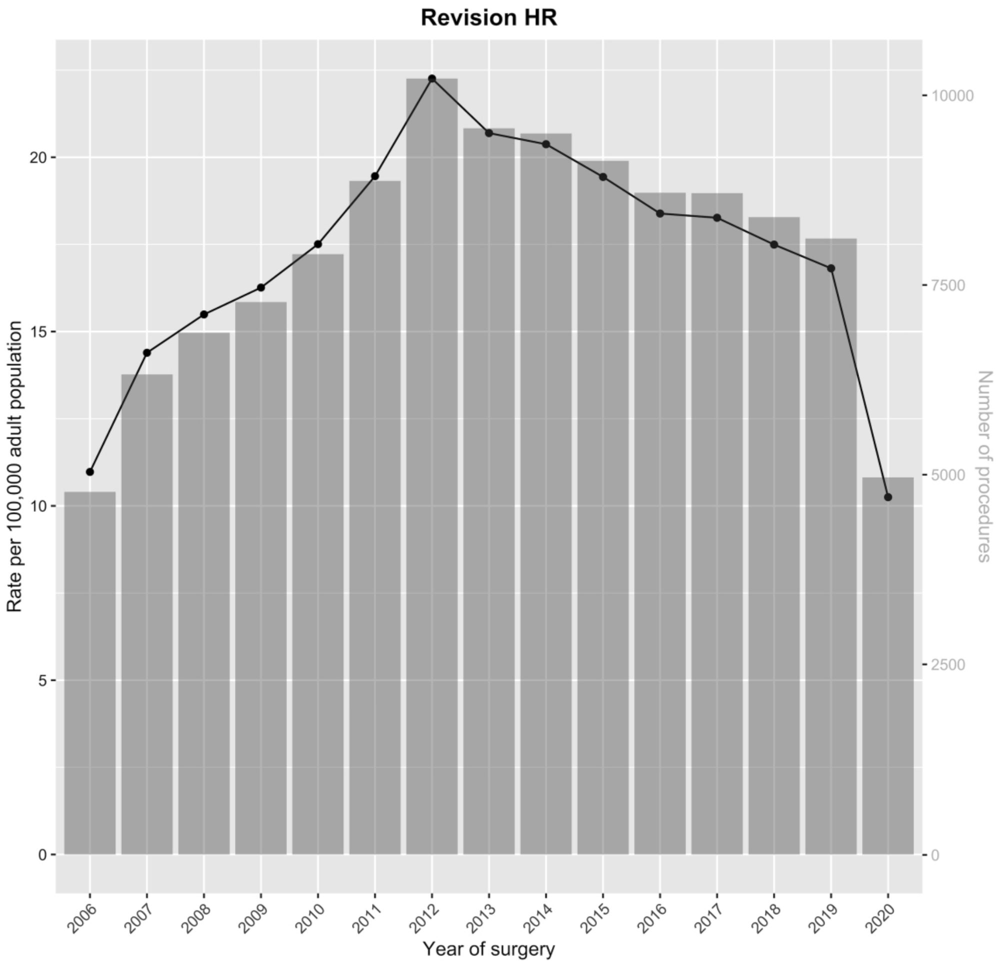

Figure 1b

Figure 1: Annual crude incidence rates (line, left y-axis) and total counts (bars, right y-axis) of (a) pHR and (b) rHR from 2006-2020. Note the differences in y-axis scales for pHR and rHR

1000x1000mm (72 x 72 DPI)

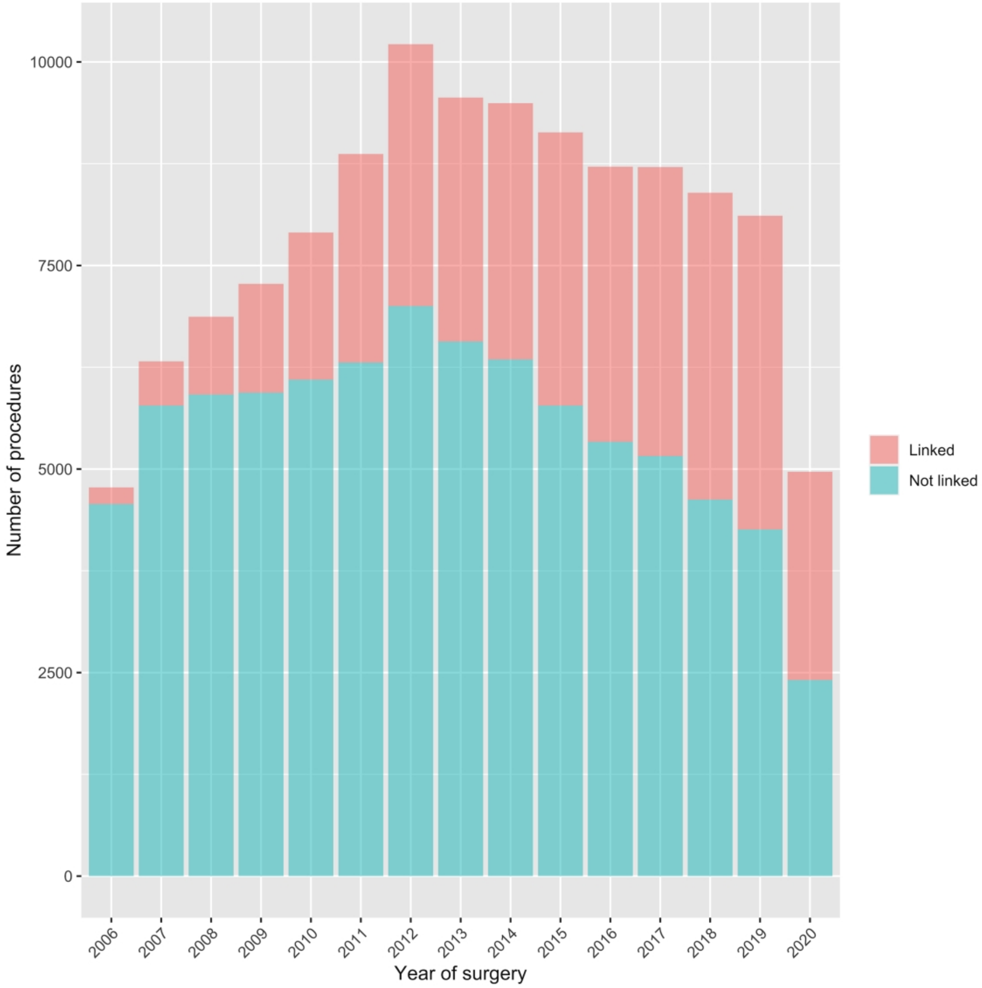

Figure 2: Changes in the annual total counts of rHR that could be linked to a pHR on the NJR over time

1000x1000mm (72 x 72 DPI)

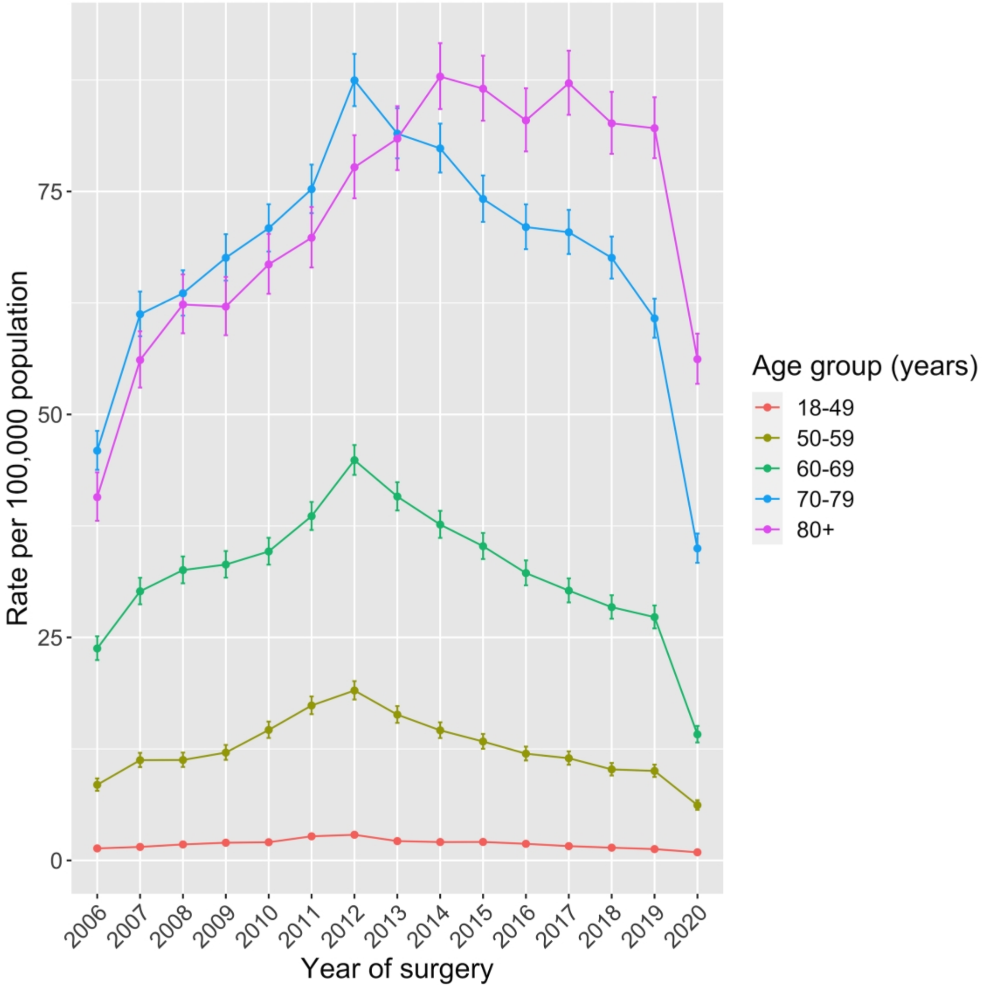

Figure 3: Annual incidence rates of first linked rHR from 2006-2020 by age group.

1000x1000mm (72 x 72 DPI)

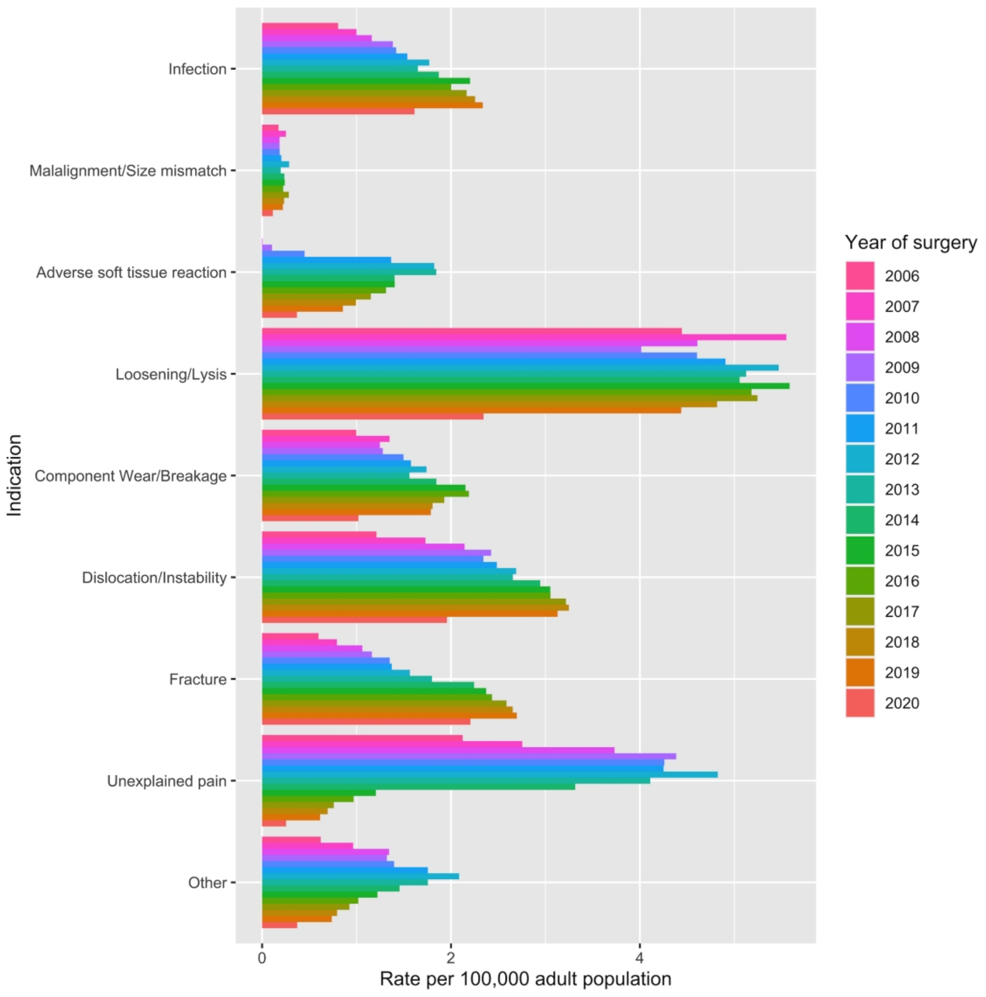

Figure 4: Grouped barplots demonstrating annual incidence rates for all rHR by indication for surgery from 2006-2020. Diagnoses are ranked in hierarchical order (greatest importance at the top).

1000x1000mm (72 x 72 DPI)

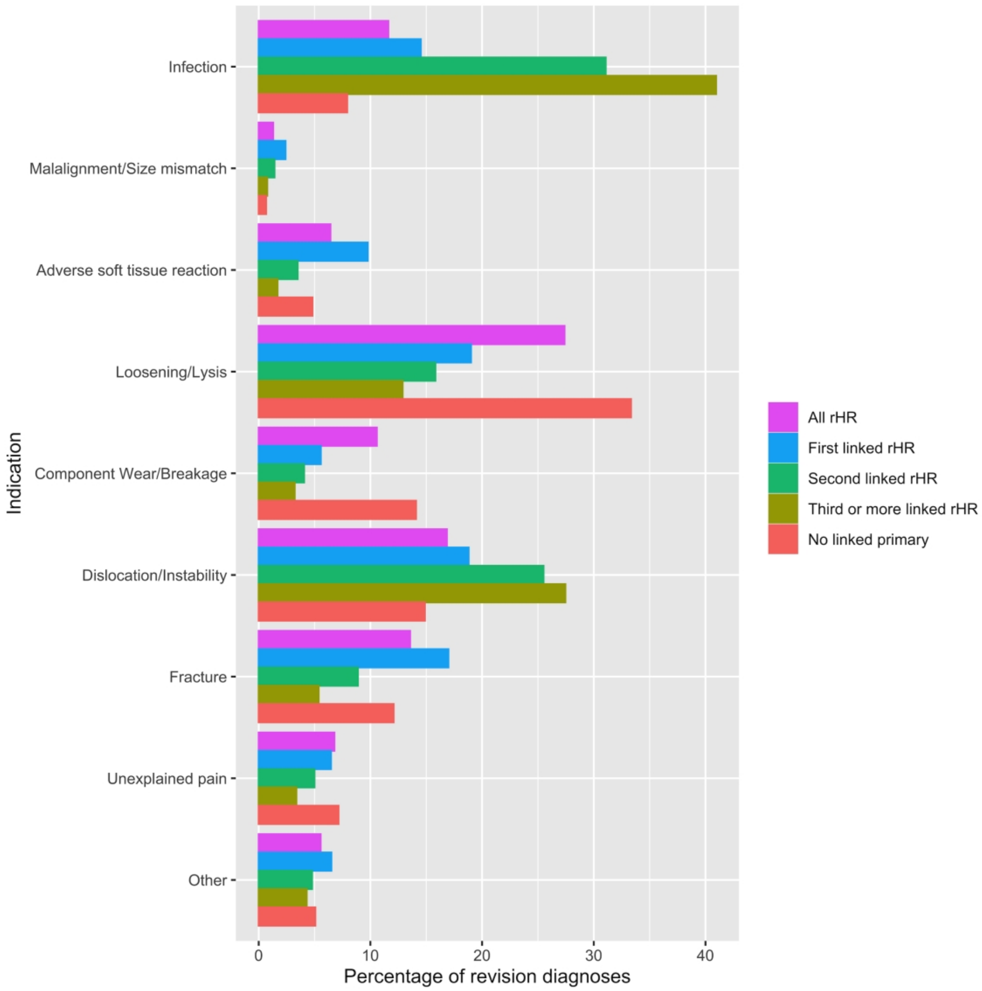

Figure 5: Percentage frequency of each indication for first and re-revision HR from 2014 to 2019. Diagnoses are ranked in hierarchical order (greatest importance at the top). For a given procedure (e.g. First linked rHR), the sum of all diagnoses is 100 percent.

1000x1000mm (72 x 72 DPI)

Supplementary Data

Appendix A – Data Cleaning

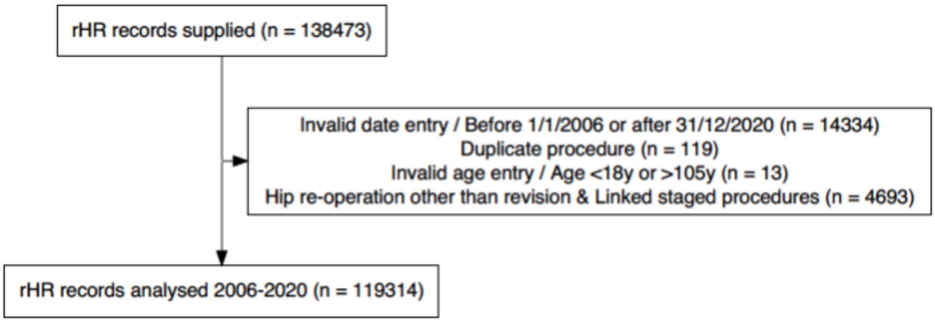

Appendix A Figure 1: Flowchart demonstrating attrition of study records during data cleaning.

peer review only

Appendix B - Methodology for calculation of directly standardised rates (DSRs) for pHR and rHR

Population estimates

The description that follows of the methodology for calculating directly standardised rates is reproduced from our recent publication [14] (permission required from Osteoarthritis and Cartilage to reproduce).

Office for National Statistics (ONS) mid-year population estimates were used to calculate changes in the usual resident population. Since Scotland has its own arthroplasty register and does not contribute cases to the NJR, we filtered out population data for Scotland. This left data for England, Wales and Northern Ireland, which - for simplicity - we have continued to refer to as the 'UK population'. A further note is that - in recent years - the NJR has started to collect cases from regions that are not part of the UK (i.e. the Isle of Man and the States of Guernsey). We have not accounted for this in mid-year population estimates and, as such, have chosen to accept a small error.

The total UK population increased in size every year over the study period, with overall growth of 10.1% from mid-2006 (55693967 persons) to mid-2019 (61333507 persons) [31]. The adult population (i.e. persons aged 18 years or older) increased by 10.9% from mid-2006 (43511312 persons) to mid-2019 (48239295 persons).

Methodology for population standardisation

Direct standardisation was performed using Byar's method with Dobson method adjustment as recommended by Public Health England [19]. We used the phe_dsr function within the PHEindicatormethods package in R for all calculations. The 2013 European Standard Population (ESP) was used as the standard population. This is a theoretical population of 100,000 persons based on European Union states' population projections for 2011 - 2030. The population structure is described in five-year age groups, where the first group is 0-4 years and the last group 90 years and older. Since it is the adult population that is relevant for pHR and rHR, we truncated and recategorised the European Standard Population into the same age groups that we had defined for the NJR (i.e. 18-49 years, 50-59 years, 60-69 years, 70-79 years, and 80+ years). To calculate the size of the 18-59 years age group, we calculated the total of all age groups from 20-24 years to 45-49 years, and then added two-fifths of the total of the 15-19 years age group. Finally, the resulting vector was transformed to provide a standard population of 100,000 persons.

Trends over time in directly standardised rates of pHR and rHR

Directly standardised rates of pHR and rHR are plotted together with crude incidence rates in Appendix B Figure 1. These rates may be useful for other countries or regions wishing to compare their rate of intervention to the UK, accounting for differences in population age structure.

For peer review only

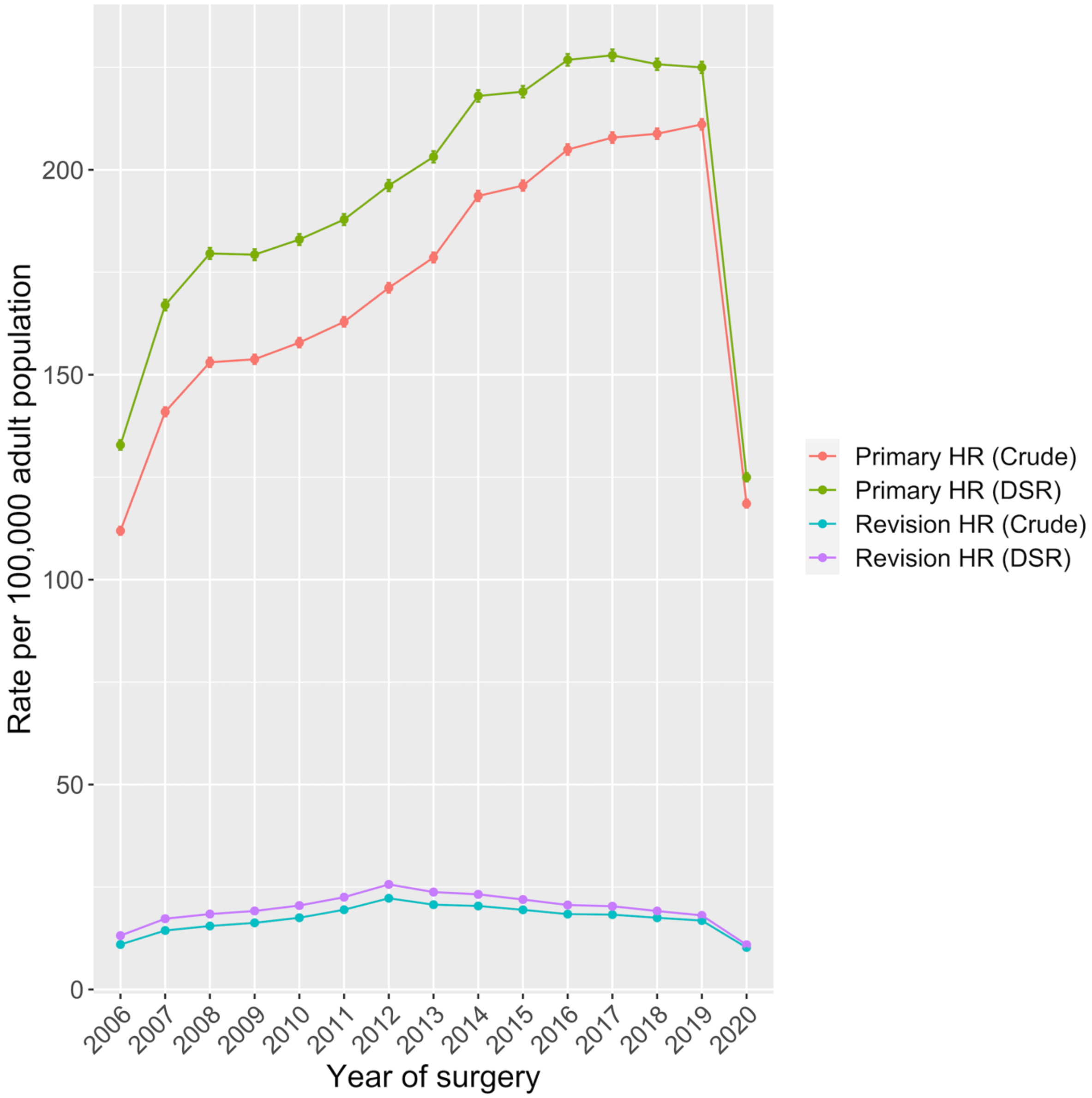

Appendix B Figure 1: Directly standardised rates (DSR) (using European Standard Population 2013) and crude rates of pHR and rHR from 2006-2020.

Appendix C - Diagnosis hierarchy for rHR

Table 2: Diagnosis hierarchy for rHR

Rank	Indication for revision	NJR indication(s) included	AOANJRR category
1	Infection	Infection	Dominant diagnosis independent of prosthesis/surgery
2	Malalignment/Size mismatch	Head/Socket Mismatch<br>Malalignment	Surgical procedure
3*	Adverse soft tissue reaction	Adverse Soft Tissue Reaction to Particulate Debris	Reaction to prosthesis
4	Loosening/Lysis	Aseptic loosening<br>Lysis	Reaction to prosthesis
5	Component wear/Breakage	Implant fracture<br>Wear of acetabular component	Wear and implant breakage
6	Dislocation/Instability	Dislocation/Subluxation<br>Dissocation of Liner	Stability of prosthesis
7	Fracture	Peri-Prosthetic Fracture	Fracture of bone
9	Unexplained pain	Unexplained pain	Pain
10	Other	Other	Remaining diagnoses

*Indication added to NJR in 2008

Table 3: Counts and percentage frequency for each indication for rHR from 2006-2019 combined

Indication for revision	n	Percent
Infection	10938	9.6
Malalignment/Size mismatch	1433	1.3
Adverse soft tissue reaction	5940	5.2
Loosening/Lysis	31796	27.8
Component Wear/Breakage	10625	9.3
Dislocation/Instability	16830	14.7
Fracture	11507	10.1
Unexplained pain	17286	15.1
Other	7993	7.0

peer review only

Appendix D - Trends over time in annual proportions of rHR by indication

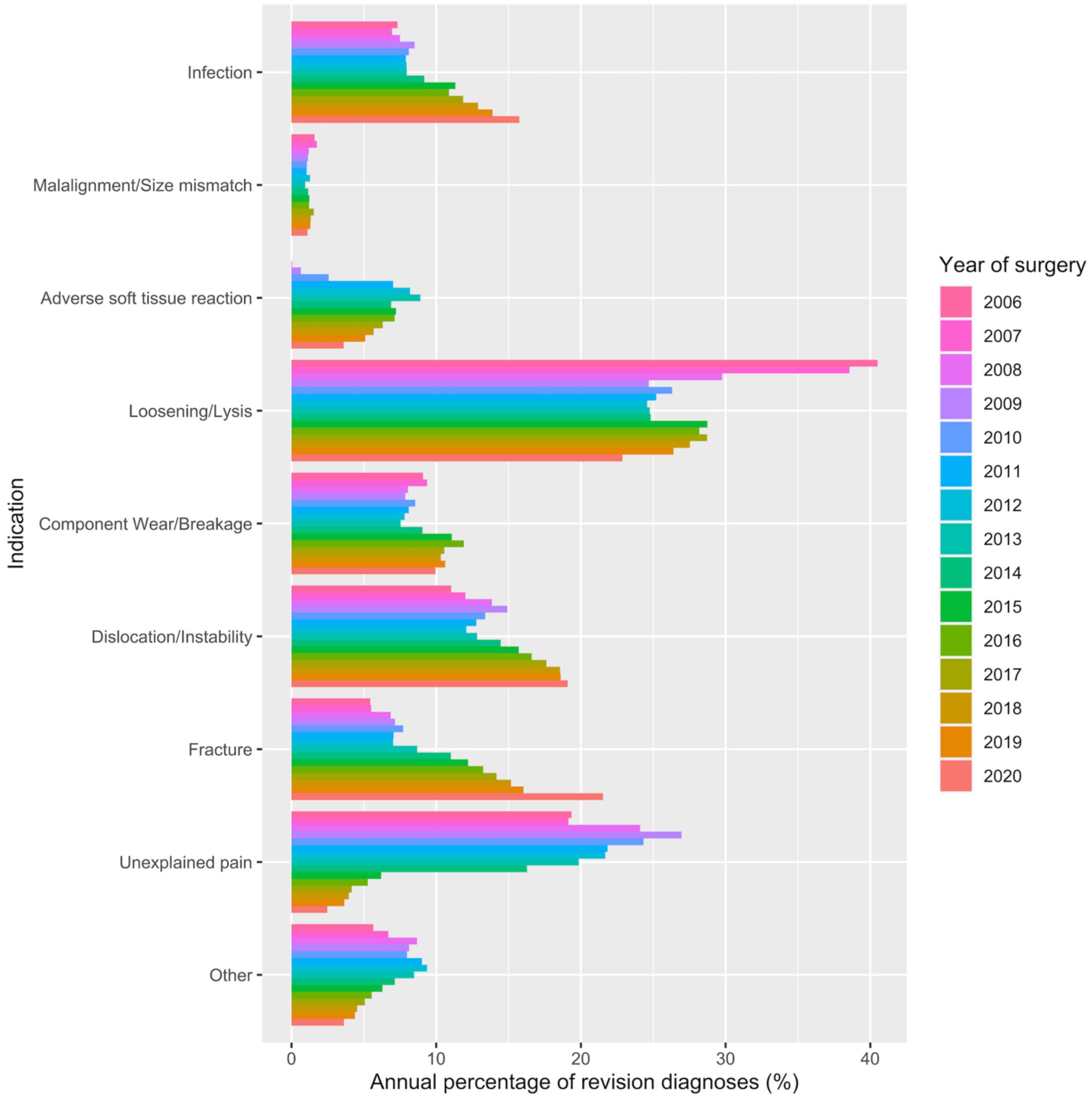

Appendix D Figure 1: Grouped barplots demonstrating changes in the annual proportions of each revision indication for all rHR from 2006-2020. Indications are ranked in hierarchical order (greatest importance at the top).

The RECORD statement – checklist of items, extended from the STROBE statement, that should be reported in observational studies using routinely collected health data.

	Item No.	STROBE items	Location in manuscript where items are reported	RECORD items	Location in manuscript where items are reported
**Title and abstract**					
	1	(a) Indicate the study's design with a commonly used term in the title or the abstract (b) Provide in the abstract an informative and balanced summary of what was done and what was found		RECORD 1.1: The type of data used should be specified in the title or abstract. When possible, the name of the databases used should be included.<br><br>RECORD 1.2: If applicable, the geographic region and timeframe within which the study took place should be reported in the title or abstract.<br><br>RECORD 1.3: If linkage between databases was conducted for the study, this should be clearly stated in the title or abstract.	Title/Abstract<br><br>Abstract<br><br>Abstract
**Introduction**					
Background rationale	2	Explain the scientific background and rationale for the investigation being reported			L58-92
Objectives	3	State specific objectives, including any prespecified hypotheses			L91-92
**Methods**					
Study Design	4	Present key elements of study design early in the paper			Abstract & L94-204
Setting	5	Describe the setting, locations, and relevant dates, including periods of recruitment, exposure, follow-up, and data collection			L116-125

For peer review only

Participants	6	*(a) Cohort study* - Give the eligibility criteria, and the sources and methods of selection of participants. Describe methods of follow-up<br>*Case-control study* - Give the eligibility criteria, and the sources and methods of case ascertainment and control selection. Give the rationale for the choice of cases and controls<br>*Cross-sectional study* - Give the eligibility criteria, and the sources and methods of selection of participants<br><br>*(b) Cohort study* - For matched studies, give matching criteria and number of exposed and unexposed<br>*Case-control study* - For matched studies, give matching criteria and the number of controls per case		RECORD 6.1: The methods of study population selection (such as codes or algorithms used to identify subjects) should be listed in detail. If this is not possible, an explanation should be provided.<br><br>RECORD 6.2: Any validation studies of the codes or algorithms used to select the population should be referenced. If validation was conducted for this study and not published elsewhere, detailed methods and results should be provided.<br><br>RECORD 6.3: If the study involved linkage of databases, consider use of a flow diagram or other graphical display to demonstrate the data linkage process, including the number of individuals with linked data at each stage.	Population selection described L116-125<br><br>NJR validation studies referenced<br><br>Figure 6
Variables	7	Clearly define all outcomes, exposures, predictors, potential confounders, and effect modifiers. Give diagnostic criteria, if applicable.		RECORD 7.1: A complete list of codes and algorithms used to classify exposures, outcomes, confounders, and effect modifiers should be provided. If these cannot be reported, an explanation should be provided.	Ref 16.
Data sources/ measurement	8	For each variable of interest, give sources of data and details of methods of assessment (measurement).<br>Describe comparability of assessment methods if there is more than one group			L127-195

For peer review only

Bias	9	Describe any efforts to address potential sources of bias			N/A
Study size	10	Explain how the study size was arrived at			All available data used
Quantitative variables	11	Explain how quantitative variables were handled in the analyses. If applicable, describe which groupings were chosen, and why			L127-195
Statistical methods	12	(a) Describe all statistical methods, including those used to control for confounding<br>(b) Describe any methods used to examine subgroups and interactions<br>(c) Explain how missing data were addressed<br>(d) *Cohort study* - If applicable, explain how loss to follow-up was addressed<br>*Case-control study* - If applicable, explain how matching of cases and controls was addressed<br>*Cross-sectional study* - If applicable, describe analytical methods taking account of sampling strategy<br>(e) Describe any sensitivity analyses			L127-195
Data access and cleaning methods		..		RECORD 12.1: Authors should describe the extent to which the investigators had access to the database population used to create the study population.	See GitHub repo

For peer review only

				RECORD 12.2: Authors should provide information on the data cleaning methods used in the study.	
Linkage		..		RECORD 12.3: State whether the study included person-level, institutional-level, or other data linkage across two or more databases. The methods of linkage and methods of linkage quality evaluation should be provided.	Person-level<br>See GitHub repo
**Results**					
Participants	13	(a) Report the numbers of individuals at each stage of the study (*e.g.*, numbers potentially eligible, examined for eligibility, confirmed eligible, included in the study, completing follow-up, and analysed)<br>(b) Give reasons for non-participation at each stage.<br>(c) Consider use of a flow diagram		RECORD 13.1: Describe in detail the selection of the persons included in the study (*i.e.,* study population selection) including filtering based on data quality, data availability and linkage. The selection of included persons can be described in the text and/or by means of the study flow diagram.	Figure 6
Descriptive data	14	(a) Give characteristics of study participants (*e.g.*, demographic, clinical, social) and information on exposures and potential confounders<br>(b) Indicate the number of participants with missing data for each variable of interest<br>(c) *Cohort study* - summarise follow-up time (*e.g.*, average and total amount)			Mostly N/A<br><br>However, see Table 1
Outcome data	15	*Cohort study* - Report numbers of outcome events or summary measures over time<br>*Case-control study* - Report numbers in each exposure			N/A

For peer review only

		category, or summary measures of exposure<br>*Cross-sectional study* - Report numbers of outcome events or summary measures			
Main results	16	(a) Give unadjusted estimates and, if applicable, confounder-adjusted estimates and their precision (e.g., 95% confidence interval). Make clear which confounders were adjusted for and why they were included<br>(b) Report category boundaries when continuous variables were categorized<br>(c) If relevant, consider translating estimates of relative risk into absolute risk for a meaningful time period			N/A
Other analyses	17	Report other analyses done—e.g., analyses of subgroups and interactions, and sensitivity analyses			
**Discussion**					
Key results	18	Summarise key results with reference to study objectives			L270-284
Limitations	19	Discuss limitations of the study, taking into account sources of potential bias or imprecision. Discuss both direction and magnitude of any potential bias		RECORD 19.1: Discuss the implications of using data that were not created or collected to answer the specific research question(s). Include discussion of misclassification bias, unmeasured confounding, missing data, and changing eligibility over time, as they pertain to the study being reported.	L307-326
Interpretation	20	Give a cautious overall interpretation of results considering objectives,			L328-337

For peer review only - http://bmjopen.bmj.com/site/about/guidelines.xhtml

		limitations, multiplicity of analyses, results from similar studies, and other relevant evidence			
Generalisability	21	Discuss the generalisability (external validity) of the study results			L328-337
**Other Information**					
Funding	22	Give the source of funding and the role of the funders for the present study and, if applicable, for the original study on which the present article is based			Title page
Accessibility of protocol, raw data, and programming code		..		RECORD 22.1: Authors should provide information on how to access any supplemental information such as the study protocol, raw data, or programming code.	GitHub repository

*Reference: Benchimol EI, Smeeth L, Guttmann A, Harron K, Moher D, Petersen I, Sørensen HT, von Elm E, Langan SM, the RECORD Working Committee. The REporting of studies Conducted using Observational Routinely-collected health Data (RECORD) Statement. *PLoS Medicine* 2015; in press.

*Checklist is protected under Creative Commons Attribution (CC BY) license.

For peer review only

BMJ Open

Epidemiology of revision hip replacement surgery in the UK over the past 15 years - An analysis from the National Joint Registry

Journal:	*BMJ Open*
Manuscript ID	bmjopen-2023-072462.R1
Article Type:	Original research
Date Submitted by the Author:	16-Aug-2023
Complete List of Authors:	Sabah, Shiraz; University of Oxford Nuffield Department of Orthopaedics Rheumatology and Musculoskeletal Sciences, Price Knee Research Group<br>Knight, Ruth; University of Oxford,<br>Nicolson, Philippa; University of Oxford, Nuffield Department of Orthopaedics, Rheumatology and Musculoskeletal Sciences<br>Taylor, Adrian; University of Oxford Nuffield Department of Orthopaedics Rheumatology and Musculoskeletal Sciences<br>Kendrick, Benjamin; University of Oxford Nuffield Department of Orthopaedics Rheumatology and Musculoskeletal Sciences<br>Alvand, Abtin; University of Oxford Nuffield Department of Orthopaedics Rheumatology and Musculoskeletal Sciences,<br>Petrou, Stavros; Nuffield Department of Primary Care Health Sciences,<br>Beard, David; University of Oxford, Nuffield Dept of Orthopaedics, Rheumatology and Musculoskeletal Sciences<br>Price, Andrew; University of Oxford, Nuffield Department of Orthopaedics, Rheumatology and Musculoskeletal Sciences<br>Palmer, Antony; University of Oxford, Nuffield Department of Orthopaedics, Rheumatology and Musculoskeletal Sciences
**Primary Subject Heading**:	Surgery
Secondary Subject Heading:	Epidemiology
Keywords:	Hip < ORTHOPAEDIC & TRAUMA SURGERY, SURGERY, Orthopaedic & trauma surgery < SURGERY

SCHOLARONE™
Manuscripts

I, the Submitting Author has the right to grant and does grant on behalf of all authors of the Work (as defined in the below author licence), an exclusive licence and/or a non-exclusive licence for contributions from authors who are: i) UK Crown employees; ii) where BMJ has agreed a CC-BY licence shall apply, and/or iii) in accordance with the terms applicable for US Federal Government officers or employees acting as part of their official duties; on a worldwide, perpetual, irrevocable, royalty-free basis to BMJ Publishing Group Ltd ("BMJ") its licensees and where the relevant Journal is co-owned by BMJ to the co-owners of the Journal, to publish the Work in this journal and any other BMJ products and to exploit all rights, as set out in our licence.

The Submitting Author accepts and understands that any supply made under these terms is made by BMJ to the Submitting Author unless you are acting as an employee on behalf of your employer or a postgraduate student of an affiliated institution which is paying any applicable article publishing charge ("APC") for Open Access articles. Where the Submitting Author wishes to make the Work available on an Open Access basis (and intends to pay the relevant APC), the terms of reuse of such Open Access shall be governed by a Creative Commons licence – details of these licences and which Creative Commons licence will apply to this Work are set out in our licence referred to above.

Other than as permitted in any relevant BMJ Author's Self Archiving Policies, I confirm this Work has not been accepted for publication elsewhere, is not being considered for publication elsewhere and does not duplicate material already published. I confirm all authors consent to publication of this Work and authorise the granting of this licence.

For peer review only

Epidemiology of revision hip replacement surgery in the UK over the past 15 years - An analysis from the National Joint Registry

Authors

1. Shiraz A. Sabah, 0000-0003-2401-1372 – shiraz.sabah@ndorms.ox.ac.uk*
2. Ruth Knight, 0000-0001-6810-2845 – ruth.knight@liverpool.ac.uk
3. Philippa Nicolson, 0000-0003-2394-4867 – philippa.nicolson@ndorms.ox.ac.uk
4. Adrian Taylor – adrian.taylor@ndorms.ox.ac.uk
5. Benjamin Kendrick – ben.kendrick@ndorms.ox.ac.uk
6. Abtin Alvand, 0000-0001-6069-978X – abtin.alvand@ndorms.ox.ac.uk
7. Stavros Petrou, 0000-0003-3121-6050 - stavros.petrou@phc.ox.ac.uk
8. David J. Beard, 0000-0001-7884-6389 – david.beard@ndorms.ox.ac.uk
9. Andrew J. Price, 0000-0002-4258-5866 – andrew.price@ndorms.ox.ac.uk
10. Antony J. R. Palmer, 0000-0003-4616-7482 – antony.palmer@ndorms.ox.ac.uk

Institutions

1. Nuffield Department of Orthopaedics, Rheumatology and Musculoskeletal Sciences, University of Oxford

2. Nuffield Orthopaedic Centre, Oxford

For peer review only

3. Liverpool Clinical Trials Centre, University of Liverpool

4. Nuffield Department of Primary Care Health Sciences, Oxford

Initials, surnames, appointments, and highest academic degree

SA Sabah: DPhil Student, BSc FRCS(Orth) 1,2*

R Knight: Senior Medical Statistician, MMath, PhD 3

P Nicolson: Post-Doctoral Researcher, BPhty, PhD 1,2

A Taylor: Consultant Orthopaedic Surgeon, FRCS(Orth) 1,2

B Kendrick: Consultant Orthopaedic Surgeon, DPhil FRCS(Orth) 1,2

A Alvand: Consultant Orthopaedic Surgeon, DPhil FRCS(Orth) 1,2

S Petrou: Professor of Health Economics, BSc MPhil PhD 4

DJ Beard: Professor of Musculoskeletal and Surgical Science, DPhil, FRCS (hon) 1

AJ Price: Professor of Orthopaedics, DPhil FRCS(Orth) 1,2

AJR Palmer: Consultant Orthopaedic Surgeon, DPhil FRCS(Orth) 1,2

Correspondence address

*Corresponding author

Price Knee Research Group, Nuffield Department of Orthopaedics, Rheumatology and Musculoskeletal Sciences (NDORMS)

Botnar Research Centre, Oxford, OX3 7LD

For peer review only

Abstract

Objectives: To investigate trends in the incidence rate and main indication for revision hip replacement (rHR) over the past 15 years in the UK.

Design: Repeated national cross-sectional study from 2006 to 2020.

Setting/Participants: rHR procedures were identified from the National Joint Registry for England, Wales, Northern Ireland, the Isle of Man and the States of Guernsey. Population statistics were obtained from the Office for National Statistics.

Main outcome measures: Crude incidence rates of rHR.

Results: The incidence rate of rHR doubled from 11 per 100,000 adults in 2006 (95% CI 10.7 to 11.3) to a peak of 22 per 100,000 adults (95% CI 22 to 23) in 2012, before falling to 17 per 100,000 adults in 2019 (95% CI 16 to 17) [24.5% decrease from peak]. The incidence rate of rHR reduced by 39% in 2020 compared with 2019 (during the Covid-19 pandemic). The most frequent indications for rHR between 2006-2019 were loosening/lysis (27.8%), unexplained pain (15.1%) and dislocation/instability (14.7%). There were incremental increases in the annual number and incidence rates of rHR for fracture, infection, dislocation/instability, and a decrease in rHR for aseptic loosening/lysis.

For peer review only

Conclusions: The incidence rate of rHR doubled from 2006 to 2012, likely due to high early failure rates of metal-on-metal hip replacements. The incidence of rHR then decreased by approximately 25% from 2012 to 2019, followed by a large decrease during the Covid-19 pandemic. The decrease in the number of rHR performed for aseptic loosening/lysis may reflect improved wear and implant longevity. Increased healthcare resource will be required to care for increased numbers of patients undergoing rHR for fracture or infection.

For peer review only

Article Summary

Strengths and limitations of this study:

- This study analysed a large national joint registry over a 15-year period to investigate trends in the rate of, and indication for, revision hip replacement (rHR).
- Crude incidence rates were calculated using arthroplasty records from the National Joint Registry and population statistics from the Office for National Statistics.
- Directly standardized rates were calculated using the 2013 European Standard Population and may facilitate international comparison of arthroplasty practices.
- Registry compliance increased over the study period. In the early years of the registry, most rHR had no associated primary hip replacement record.
- The analysis of re-revision procedures over-represents early modes of failure (such as infection or fracture) and under-represents late modes of failure (such as aseptic loosening/osteolysis and component wear).

Word count: 3264 words

Keywords: total hip replacement; revision; re-operation; epidemiology

For peer review only

Introduction

Primary hip replacement (pHR) is a highly successful intervention and is associated with large improvements in joint function and health-related quality of life (HRQoL) [1]. Prior to the Covid-19 pandemic, most international joint replacement registries and insurance databases reported increasing counts of pHR each year [2–4]. Nearly all analyses project an increase in the requirement for pHR over the coming decades [5–7], due to ageing populations and associated increases in the prevalence of hip osteoarthritis [8,9].

Revision hip replacement (rHR) refers to a heterogeneous group of surgical procedures that usually involve the exchange or modification of one or more components of an existing hip replacement. It is performed infrequently compared with pHR and, following a pHR most patients do not require further surgery to that hip during their lifetime [10,11]. However, increases in the number of pHR performed each year, and in younger patients, coupled with greater life expectancy, results in an increase in the number of pHR that are at risk of failure. This is expected to create a large increase in the number of rHR procedures required in the future [5]. The epidemiology and trends in rHR are not well understood.

The National Joint Registry (NJR) for England, Wales, Northern Ireland, the Isle of Man and the States of Guernsey provides information on annual total counts of rHR [12]. This registry data has shown that the total number of rHR in the UK peaked in 2012 and has since declined. This observation is thought to be due to the high utilisation of metal-on-metal hip replacements (MOM-HR) either side of the turn of the 21st century, some of which had very high, early failure rates

For peer review only

111 [12]. Count data provide only a limited perspective. They are useful for understanding the number
112 of surgeons and surgical units performing rHR, which may, for example, inform budget estimates.
113 However, they do not provide information on the rate of intervention, or trends in different
114 procedures and patient groups, which requires knowledge of the size and demographics of the
115 general population. Count data are also of limited value for comparing UK practice with other
116 countries. It is also important to understand whether the indications for rHR have temporally
117 changed. In revision knee replacement (rKR), the number and proportion of procedures performed
118 for infection have increased, whilst procedures for aseptic loosening have decreased [13,14]. It is
119 currently unknown whether a similar trend exists for rHR. The aim of our study was to investigate
120 trends in the incidence rHR by indication during the past fifteen years in the UK, including the
121 impact of the COVID-19 pandemic.

For peer review only

Patients and Methods

This study was reported according to the RECORD checklist [15].

Study dataset

The NJR prospectively collects data on pHR and rHR from hospital providers within its geographic remit. Data submission has been mandatory for independent sector providers since 2003 and for NHS providers since April 2011 [16]. rHR procedures are submitted to the NJR using H2 Minimum Data Set forms, which include fields on patient, surgeon, operation and implant characteristics. The current NJR definition of revision is "any operation where one or more components are added to, removed from or modified in a joint replacement or if a Debridement And Implant Retention (DAIR) with or without modular exchange is performed". The main change to this definition over the study period was the inclusion of DAIR procedures without modular exchange from 25/06/2018. Further information on all amendments to this definition can be found in the glossaries of NJR annual reports and the publication Operations included in the NJR [17].

Timeframe

This study examined NJR records for rHR performed between 1st January 2006 and 31st December 2020. We chose to exclude NJR records from 2003 to 2005, due to the known poor compliance with the registry when first formed [18,19]. Records from 2021 were excluded because: (i) the NJR data extract was produced on 3rd February 2022, which meant a limited

For peer review only

window for late submission of data; and (ii) population statistics from the Office for National Statistics (ONS) were unavailable. We analysed longer-term trends using records from 2006 to 2019. We hypothesised that rHR activity in 2020 was unlikely to be representative of usual practice due to the disruption in arthroplasty services as a result of the Covid-19 pandemic. To measure the impact of the Covid-19 pandemic, we compared data from 2019 (pre-pandemic) to 2020 (pandemic).

Statistics

All statistical code is available from the lead author's GitHub page.

Trends over time in the incidence rate of rHR

Crude incidence rates of pHR and rHR were calculated for each calendar year from 2006 to 2020. pHR was defined as any procedure reported to the NJR using a H1 form. This included total hip replacement and hip resurfacing procedures performed for any indication, where both an acetabular and a femoral component were implanted. The crude incidence rate was defined as the annual total count of procedures in a calendar year divided by the sum of the mid-year population estimates for adults in England, Wales and Northern Ireland. The *phe_rate* function within the PHEindicatormethods package in R was used to calculate 95% confidence intervals following Byar's method [20,21]. In some situations, a rHR may be performed as a staged procedure with two distinct operations on different days. We considered first- and second-stage rHR as a single

For peer review

procedure to calculate incidence rates. The following criteria were applied to bundle these procedures: (i) The NJR H2 form completed at the first-stage procedure was required to specify the intention to treat the patient in stages, and (ii) the second-stage procedure was required to have been performed within 365 days of the first-stage. Analysis of hospital resource utilisation should count each stage individually, however, since these totals are readily available in NJR annual reports we did not reproduce this analysis [2]. The crude incidence rate was presented as a line graph overlying bars of annual total counts. Long term trends in total counts and incidence rates were analysed descriptively from 2006 to 2019. The percentage decrease in the total count of rHR during the Covid pandemic was analysed as the change in total count from 2019 to 2020, divided by the total count in 2019. The percentage decrease in the incidence rate of rHR was calculated following the same principles.

We investigated trends over time in the number of rHR procedures linked to a pHR on the NJR. This analysis is presented as stacked bars of annual total counts for linked and not linked procedures. Our dataset did not allow us to compare incidence rates for first and subsequent re-revision procedures because the proportion of linked procedures changed markedly over the study period. For the same reason, we were unable to perform any meaningful analysis of first-linked rHR by pHR bearing surface type (for example, MOM versus other types). The incidence rate of rHR within a given year is not strongly associated with the incidence rate of pHR in that year, because few pHR fail within one year . The prevalence of pHR and rHR in the general population and the rate of failure of these implants is directly related to the incidence of rHR. However, we decided not to model prevalence due to the significant uncertainty around these estimates, given the longevity of hip replacements[11] and the comparatively short existence of the NJR. To enable

For peer review only

future comparisons to populations with different age-structures (for example, from different countries around the world) we calculated directly standardised rates (DSRs) for pHR and rHR. This methodology is described in Appendix A (Supplementary File) and uses the 2013 European Standard Population.

Trends over time in the incidence rate of rHR by patient characteristics

The study population was stratified into the following groups to investigate trends over time in the incidence rate of rHR by age at the time of revision surgery: 18-49 years/ 50-59 years/ 60-69 years/ 70-79 years/ 80+ years. We considered these groupings to fairly represent the data and to be interpretable, after exploring different categorizations. Age-specific incidence rates per 100,000 persons were calculated for each group for each year of analysis and presented graphically. Each group was further stratified into female and male to calculate age- and gender-specific incidence rates. Further analyses were performed to investigate trends over time in American Society of Anesthesiologists (ASA) classification and Body Mass Index (BMI).

Trends in the main indication for rHR

Each rHR procedure was assigned a single, dominant indication for surgery using a diagnosis hierarchy based on the Australian Orthopaedic Association National Joint Replacement Registry (AOANJRR) model [22] (Appendix B Table 1, Supplemental Table). Pre-pandemic data (2006 to 2019) were combined to calculate the percentage frequency of each indication. To investigate

For peer review only

trends over time, crude incidence rates per 100,000 adults and annual percentage frequencies were calculated for each indication in each year (from 2006 to 2020, i.e. including pandemic data). These data were presented using grouped barplots.

To investigate the proportional share of each revision indication in first- and subsequent re-revision rHR, we analysed data from the five years immediately pre-pandemic (2014 to 2019). We excluded earlier NJR data as we considered it to be most vulnerable to the maturity of the registry. We defined the following groups: first linked rHR (the earliest revision procedure for a given patient-side linked to a primary hip replacement on the NJR); second linked rHR (the next revision procedure for a given patient-side linked to a first linked rHR); third or more linked rHR (subsequent revision procedure(s) linked to a second linked rHR); and no linked primary (revision procedure(s) not linked to a primary procedure). These data were presented using barplots.

Software

Statistical analyses were performed using R version 4.2.1.

Patient and Public Involvement

None.

For peer review only

Results

Data cleaning and linkage

The attrition of study records during data cleaning is illustrated in the Supplemental Figure Appendix C Figure 1.

Trends over time in the incidence rate of rHR

Annual total counts and crude incidence rates of rHR procedures reported to the NJR are presented in Figure 1 and Supplemental Table Appendix D Table 1. Annual total counts increased every year from 2006 (4775 rHR) to 2012 (10,217 rHR) [114% increase]. Following this peak, annual total counts declined, with 8111 rHR performed in 2019 [20.6% decrease from peak]. The incidence rate of rHR increased from 11.0 per 100,000 adults in 2006 (95% CI 10.7 to 11.3) to 22.3 per 100,000 adults in 2012 (95% CI 21.8 to 22.7), before falling to 16.8 per 100,000 adults in 2019 (95% CI 16.5 to 17.2) [24.5% decrease from peak]. In 2020, where rHR practice was affected by the Covid-19 pandemic, 4966 rHR procedures were reported to the NJR: a crude incidence rate of 10.2 per 100,000 adults (95% CI 10.0 to 10.5). These figures represented a 38.8% reduction in the total count of rHR and a 39.0% reduction in the incidence rate of rHR in 2020 compared to 2019. The percentage of rHR procedures linked to a pHR on the NJR increased from 4.3% in 2006 to 47.5% in 2019 (rising further to 51.5% in 2020) (Figure 2). The annual total counts of pHR increased every year from 2006 (48,700 pHR) to 2019 (101,828 pHR). However, increases were smaller during the last four years. The incidence rate of pHR increased from 112 per 100,000 adults in 2006 (95% CI 111 to 113) to 211 per 100,000 adults in 2019 (95% CI 210 to 212). This represented a 109.1% increase in the total count and 88.6% increase in the incidence rate of pHR

For peer review only

from 2006 to 2019. In 2020, during the Covid-19 pandemic, 57,437 pHR procedures were reported to the NJR: a crude incidence rate of 119 per 100,000 adults (95% CI 118 to 120). This represented a 43.6% reduction in the total count of pHR and a 43.8% reduction in the incidence rate of pHR in 2020 compared to 2019.

Trends over time in the incidence rate of rHR by patient characteristics

The incidence rate of rHR increased in all age groups from 2006 to 2012 (Figure 3). The group with the largest increase in the incidence rate of rHR over this period was patients aged 70-79 years at the time of surgery, from 46 per 100,000 adults in 2006 [95% CI 44 to 48] to 87 per 100,000 adults in 2012 [95% CI 85 to 90]. The incidence rate of rHR declined for all age groups from 2012 to 2019, except patients aged 80 years or older at the time of surgery. In this patient group, the incidence rate of rHR peaked in 2014 (88 per 100,000 adults [95% CI 84 to 92]) and remained relatively unchanged through to 2019. Age- and gender-specific incidence rates for all rHR are provided in Supplemental Figure Appendix D Figure 1. The rate of intervention was generally higher in female patients, though differences were smaller in more recent years. The analysis of patient's overall health found that the proportion of patients who were ASA Class 3+ increased over the study period, from 25.3% in 2006 to 45.8% in 2019, with a further increase in 2020 (49.4%). Both ASA Class 1 and ASA Class 2 groups became smaller over the study period (Supplemental Figure Appendix D Figure 2). The analysis of trends in BMI were difficult to interpret due to the large proportion of missing data, though this fraction reduced over the study period (Supplemental Figure Appendix D Figure 3).

Trends in the main indication for rHR

For peer review only

During the years 2006 to 2019 combined, aseptic loosening was the most frequent indication for rHR (27.8%), followed by unexplained pain (15.1%) and then dislocation/instability (14.7%) (Supplemental Table Appendix B Table 2). The incidence rate of rHR for infection increased nearly every year from 2006 (0.8 per 100,000 adults [95% CI 0.7 to 0.9]) to 2019 (2.3 per 100,000 adults [95% CI 2.2 to 2.5]) (Figure 4). A similar trend was observed for rHR for dislocation/instability and fracture. The incidence rate for malalignment/size mismatch changed little over the study period (~0.2 per 100,000 adults). For the remaining indications, incidence rates increased to a peak before decreasing in more recent years. rHR for adverse soft tissue reaction, unexplained pain, and 'other' peaked in 2012 and 2013. rHR for loosening/lysis and component wear/breakage peaked later, in 2015 and 2016 respectively.

Trends in the annual *proportional share* of each revision indication are presented in the supplementary material (Supplemental Figure Appendix D Figure 4). These data should be interpreted in conjunction with crude incidence rates. For example, whilst the proportion of rHR for loosening/lysis was lower in 2019 compared to 2006, the crude incidence rate was greater.

The proportion of revisions attributable to each indication within first-linked rHR and re-revision rHR from 2014-2019 is demonstrated in Figure 5. A linked pHR could not be found on the NJR for the majority of rHR performed over this period (40.1% [n= 21053/52554] linked). Among the rHR that could not be linked to a pHR, loosening/lysis was the most frequent diagnosis (33.3%, n= 10,500/31,501). Within first-linked rHR, loosening/lysis (19%, n= 3448/18138) and dislocation/instability (18.8%, n= 3409/18138)) were the most frequent diagnoses. Within linked

For peer review only

re-revision rHR, infection was the most frequent diagnosis (31.1% second linked rHR, n= 703/2263; and 41.0% third or more linked rHR, n= 267/652).

For peer review only

Discussion

The total number and incidence rate of rHR doubled from 2006 to 2012, likely driven by high early failure rates of some types of MOM-HR. The incidence rate of rHR then fell in subsequent years, and was three-quarters of its peak by 2019, suggesting improved pHR longevity. The total number and incidence rate of pHR reported to the NJR approximately doubled from 2006 to 2019, with increases every year until the Covid-19 pandemic. In 2020, during the Covid-19 pandemic, approximately 40% fewer rHR were performed compared with 2019. Patients aged 80 years and older displayed the largest increase in the rate of rHR during the study period. Contrary to younger age groups, where the count and rate of rHR procedures decreased after 2012, rHR activity in this older patient group was maintained. Between the years 2006 and 2019, aseptic loosening was the most frequent indication for rHR (27.8%), followed by unexplained pain (15.1%) and then dislocation/instability (14.7%). There were incremental increases in annual counts and incidence rates of rHR for infection, dislocation/instability and fracture over the study period, whereas rHR for loosening/lysis decreased in absolute and proportional terms.

Our findings differ from previous studies that have forecast progressive increases in rHR activity [5,23,24]. The peak of rHR in 2012 is likely to reflect high early failure rates of MOM-HR prostheses. Many primary MOM-HR predate the NJR. The increase in the incidence of rHR for adverse soft tissue reaction up until 2012/13 and subsequent decrease in this indication supports this conclusion. One caveat to note that this indication was only added to the H2 MDS form in 2008 and is not exclusive to MOM-HR. Loosening/lysis was the most frequent indication for rHR overall, and for each year studied except 2009, where it was unexplained pain. We observed a

For peer review only

decrease in the rate and proportion of rHR for loosening/lysis in the most recent years studied, which may represent low wear rates and improved longevity of modern implants. Similar to other studies, we observed large increases in rHR for infection, fracture and instability over the study period [24].

This study is the first to provide crude incidence rates, age-specific rates and directly standardised rates for rHR in the UK using NJR data. The strengths of this study include its reproducibility and openness. The study report was written in RMarkdown with all results derived from the source data, and statistical code made available to readers. However, the study does have limitations. Our study design is not able to differentiate between changes in reporting and changes in surgical practice. There is evidence that compliance with the NJR increased over the study period [2,18,19], which suggests that the true decrease in rHR in recent years may be greater than our estimates. The hierarchy we have used to assign a single, dominant indication for rHR may not reflect the true diagnosis in all cases. The coding of indications for surgery was modified over the study period (for example, 'unexplained pain' was originally termed 'pain' on data collection forms). The indications reported to the NJR reflect those thought to apply at the time of surgery. In the case of infection, which is sometimes diagnosed post-operatively, there is evidence to suggest it may be under-recognised by registries [25]. Procedures that are not considered a revision procedure - for example, operative fixation of a periprosthetic fracture, were not included in the study. There is continued uncertainty whether periprosthetic hip fractures should be managed with revision or fixation, and trends have changed over time [26,27]. Our subgroup analysis of first-linked, second-linked and third or more-linked rHR excluded procedures with no pHR recorded in the registry. It is likely that our analyses of re-revision procedures over-represent failure modes

For peer review only

occurring in early follow-up (such as infection or fracture) and under-represent failure modes more likely to occur at later timepoints (such as aseptic loosening/osteolysis and component wear) [28]. We attempted to mitigate this effect by restricting our analysis to more mature registry data (2014 to 2019), but longer-term data are required. We have not projected future requirements for rHR.

Our study demonstrates the considerable disruption to rHR practice during the Covid-19 pandemic, similar to trends recently reported for pHR, pKR and rKR [14,29]. In the near future, the number of rHR performed is likely to increase to fulfil the backlog of patients whose care was delayed during the Covid-19 pandemic [30]. It is important to consider that the smaller than expected increases in rHR may be due to limited capacity in the healthcare system to deliver more of these procedures. There are currently major infrastructure projects in the UK to increase capacity for both primary and revision joint replacement procedures. These include development of surgical hubs to separate elective and emergency care, with the idea that planned surgical activity may continue with fewer disruptions during surges of unplanned hospital admissions [31]. Over the next few years, it will be important to measure the effectiveness of these programmes. Our study has also investigated trends over time in the main indication for rHR. The recent decreases we observed in rHR for loosening/lysis and adverse soft tissue reaction may reflect greater use of highly-rated implants [32]. Similarly, decreases in rHR for ‘unexplained pain’ may suggest that evidence-based indications for revision surgery are being followed. These may mitigate the future revision burden. In contrast, if current trends continue, fracture, infection and instability will represent an increasing proportion of rHR procedures. This has implications for healthcare provision since these indications are associated to greater complication rates, costs and utilisation

For peer review only

368 of hospital resources in comparison to elective, aseptic revision joint replacement procedures [33–
369 36].

370

371 In conclusion, the incidence rate of rHR doubled from 2006 to 2012, likely due to high early failure
372 rates of metal-on-metal hip replacements. The incidence of rHR decreased by approximately 25%
373 from 2012 to 2019, likely due to increased implant longevity. Recent trends suggest a rise in rHR
374 for fracture, infection, and dislocation/instability, and a fall in rHR for loosening/lysis. There was
375 a large reduction in the incidence of rHR during the Covid-19 pandemic in 2020.

376

377

For peer review only

Figure legends

Figure 1: Annual crude incidence rates (line, left y-axis) and total counts (bars, right y-axis) of (a) pHR and (b) rHR from 2006-2020. Note the differences in y-axis scales for pHR and rHR.

Figure 2: Changes in the annual total counts of rHR that could be linked to a pHR on the NJR over time

Figure 3: Annual incidence rates of first linked rHR from 2006-2020 by age group.

Figure 4: Grouped barplots demonstrating annual incidence rates for all rHR by indication for surgery from 2006-2020. Diagnoses are ranked in hierarchical order (greatest importance at the top).

Figure 5: Percentage frequency of each indication for first and re-revision HR from 2014 to 2019. Diagnoses are ranked in hierarchical order (greatest importance at the top). For a given procedure (e.g. First linked rHR), the sum of all diagnoses is 100 percent.

For peer review only

Contributorship statement

All authors made substantial contributions to the work. CRediT roles:

- SAS: Conceptualisation, Data curation, Formal analysis, Funding acquisition, Investigation, Methodology, Software, Writing - original draft
- RK: Data curation, Formal analysis, Statistical expertise, Investigation, Methodology, Software, Writing - review & editing
- PN: Investigation, Methodology, Software, Writing - review & editing
- AT: Investigation, Writing - review & editing
- BK: Investigation, Writing - review & editing
- AA: Investigation, Writing - review & editing
- SP: Investigation, Writing - review & editing
- DJB: Conceptualisation, Funding acquisition, Investigation, Methodology, Supervision, Writing - review & editing
- AJP: Conceptualisation, Funding acquisition, Investigation, Methodology, Supervision, Writing - review & editing
- AJRP: Conceptualisation, Investigation, Methodology, Supervision, Writing - review & editing

For peer review only

Competing interests

SAS is funded by an NIHR Doctoral Research Fellowship, Royal College of Surgeons One Year Fellowship and funding from the Rosetrees Trust, and is a member of the editorial board of the Bone & Joint Journal. AA has received funding from Zimmer Biomet and Medacta International outside of the submitted work. AJP has received funding from Zimmer Biomet outside of the submitted work and is a board member of the British Orthopaedic Association and British Association for Surgery of the Knee. BK has received funding from Adler Ortho outside of the submitted work. SP is an NIHR Senior Investigator. DJB has received institutional funding from the NIHR and Rosetrees Trust.

Funding

This study received funding from the National Institute of Health Research (Doctoral Research Fellowship 301771), Royal College of Surgeons of England (Grant no. N/A) and Rosetrees Trust (Grant no. N/A. None of the funding sources had any role in the design or conduct of the study.

Data sharing statement

Data is available from the National Joint Registry following approval by their Research Subcommittee. Statistical code is available as a repository on the lead author's GitHub page.

Ethics approval statement

For peer review only

Ethical approval was obtained from the London-Bromley Research Ethics Committee (20/LO/0428). Data access approvals were obtained from the National Joint Registry (NJR) for England, Wales, Northern Ireland, the Isle of Man and the States of Guernsey (RSC2017/26). Data for patients who did not to consent to the NJR audit were not included.

For peer review only

Acknowledgements

We thank the patients and staff of all the hospitals who have contributed data to the National Joint Registry. We are grateful to the Healthcare Quality Improvement Partnership (HQIP), the NJR Research Committee and staff at the NJR for facilitating this work. The authors have conformed to the NJR's standard protocol for data access and publication. The views expressed represent those of the authors and do not necessarily reflect those of the National Joint Registry Steering Committee, Research Sub-committee or the Healthcare Quality Improvement Partnership (HQIP) who do not vouch for how the information is presented.

For peer review only

References

1 Sabah SA, Alvand A, Beard DJ, *et al.* Minimal important changes and differences were estimated for Oxford hip and knee scores following primary and revision arthroplasty. *Journal of Clinical Epidemiology* 2022;**143**:159–68. doi:10.1016/j.jclinepi.2021.12.016

2 National Joint Registry for England Wales Northern Ireland and the Isle of Man. NJR 18th Annual Report. Published Online First: 2021.https://www.njrcentre.org.uk/njrcentre/Reports-Publications-and-Minutes/Annual-reports (accessed 16 Oct 2021).

3 Australian Orthopaedic Association. Hip, Knee & Shoulder Arthroplasty Annual Report. Published Online First: 2021.https://aoanjrr.sahmri.com/annual-reports-2021 (accessed 29 Sep 2022).

4 Swedish Arthroplasty Register. Annual Report 2021. Published Online First: 2021.https://registercentrum.blob.core.windows.net/slr/r/SAR-Annual-Report-2021-SJlAFmlRl5.pdf (accessed 30 Sep 2022).

5 Kurtz S, Ong K, Lau E, *et al.* Projections of Primary and Revision Hip and Knee Arthroplasty in the United States from 2005 to 2030. *The Journal of Bone and Joint Surgery (American)* 2007;**89**:780. doi:10.2106/JBJS.F.00222

6 Singh JA, Yu S, Chen L, *et al.* Rates of Total Joint Replacement in the United States: Future Projections to 2020–2040 Using the National Inpatient Sample. *The Journal of Rheumatology* 2019;**46**:1134–40. doi:10.3899/jrheum.170990

7 Matharu G, Culliford D, Blom A, *et al.* Projections for primary hip and knee replacement surgery up to the year 2060: An analysis based on data from The National Joint Registry for England, Wales, Northern Ireland and the Isle of Man. *The Annals of The Royal College of Surgeons of England* Published Online First: December 2021. doi:10.1308/rcsann.2021.0206

8 Spitaels D, Mamouris P, Vaes B, *et al.* Epidemiology of knee osteoarthritis in general practice: A registry-based study. *BMJ Open* 2020;**10**:e031734. doi:10.1136/bmjopen-2019-031734

9 McKee M, Dunnell K, Anderson M, *et al.* The changing health needs of the UK population. *The Lancet* 2021;**397**:1979–91. doi:https://doi.org/10.1016/S0140-6736(21)00229-4

10 Bayliss LE, Culliford D, Monk AP, *et al.* The effect of patient age at intervention on risk of implant revision after total replacement of the hip or knee: A population-based cohort study. *The Lancet* 2017;**389**:1424–30. doi:10.1016/S0140-6736(17)30059-4

11 Evans JT, Evans JP, Walker RW, *et al.* How long does a hip replacement last? A systematic review and meta-analysis of case series and national registry reports with more than 15 years of follow-up. *Lancet (London, England)* 2019;**393**:647–54. doi:10.1016/S0140-6736(18)31665-9

12 National Joint Registry for England Wales Northern Ireland and the Isle of Man. NJR 19th Annual Report. Published Online First: 2022.https://reports.njrcentre.org.uk/downloads (accessed 29 Sep 2022).

13 Lewis PL, Graves SE, Robertsson O, *et al.* Increases in the rates of primary and revision knee replacement are reducing: A 15-Year registry study across 3 continents. *Acta Orthopaedica* 2020;**91**:414–9. doi:10.1080/17453674.2020.1749380

14 Sabah SA, Knight R, Alvand A, *et al.* No exponential rise in revision knee replacement surgery over the past 15 years: An analysis from the National Joint Registry. *Osteoarthritis and Cartilage* Published Online First: 2022. doi:10.1016/j.joca.2022.08.016

15 Benchimol EI, Smeeth L, Guttmann A, *et al.* The REporting of studies Conducted using Observational Routinely-collected health Data (RECORD) Statement. *PLOS Medicine* 2015;**12**:e1001885. doi:10.1371/journal.pmed.1001885

16 Boulton C, Harrison C, Wilton T, *et al.* Implementing large-scale data quality validation in a national arthroplasty registry to improve compliance. *Bone & Joint Open* 2022;**3**:716–25. doi:10.1302/2633-1462.39.BJO-2022-0051.R1

17 National Joint Registry for England Wales Northern Ireland, the Isle of Man and the States of Guernsey. Operations included in the NJR - Version 8. 2018. https://www.njrcentre.org.uk/wp-content/uploads/Operations-included-in-NJR-v8.pdf (accessed 23 Jun 2022).

18 Sabah SA, Henckel J, Cook E, *et al.* Validation of primary metal-on-metal hip arthroplasties on the National Joint Registry for England, Wales and Northern Ireland using data from the London Implant Retrieval Centre: A study using the NJR dataset. *The bone & joint journal* 2015;**97-B**:10–8. doi:10.1302/0301-620X.97B1.35279

19 Sabah SA, Henckel J, Koutsouris S, *et al.* Are all metal-on-metal hip revision operations contributing to the National Joint Registry implant survival curves? *Bone and Joint Journal* 2016;**98B**:33–9. doi:10.1302/0301-620X.98B1.36431

20 Public Health England. Technical guide: Confidence Intervals. Published Online First: May 2018.https://fingertips.phe.org.uk/documents/PHDS%20Guidance%20-%20Confidence%20Intervals.pdf (accessed 19 Nov 2021).

For peer review only

21 Georgina A, Sebastian F, Matthew F, *et al.* PHEindicatormethods: Common Public Health Statistics and their Confidence Intervals. 2020.https://CRAN.R-project.org/package=PHEindicatormethods (accessed 18 Dec 2021).

22 Graves S, Davidson D, de Steiger R, *et al.* Australian Orthopaedic Association National Joint Replacement Registry Annual Report 2010. *Sydney, Australia: Australian Medical Association* 2009;**1914**:1–200.

23 Patel A, Pavlou G, Mújica-Mota RE, *et al.* The epidemiology of revision total knee and hip arthroplasty in England and Wales: A comparative analysis with projections for the United States. a study using the national joint registry dataset. *Bone and Joint Journal* 2015;**97-B**:1076–81. doi:10.1302/0301-620X.97B8.35170

24 Schwartz AM, Farley KX, Guild GN, *et al.* Projections and Epidemiology of Revision Hip and Knee Arthroplasty in the United States to 2030. *The Journal of Arthroplasty* 2020;**35**:S79–85. doi:10.1016/j.arth.2020.02.030

25 Sinagra ZP, Davis JS, Lorimer M, *et al.* The accuracy of reporting of periprosthetic joint infection to the Australian Orthopaedic Association National Joint Replacement Registry. *Bone & Joint Open* 2022;**3**:367–74. doi:10.1302/2633-1462.35.BJO-2022-0011.R1

26 Maggs JL, Swanton E, Whitehouse SL, *et al.* B2 or not B2? That is the question: a review of periprosthetic fractures around cemented taper-slip femoral components. *The Bone & Joint Journal* 2021;**103-B**:71–8. doi:10.1302/0301-620X.103B1.BJJ-2020-0163.R1

27 Powell-Bowns MFR, Oag E, Ng N, *et al.* Vancouver B periprosthetic fractures involving the Exeter cemented stem. *The Bone & Joint Journal* 2021;**103-B**:309–20. doi:10.1302/0301-620X.103B2.BJJ-2020-0695.R1

28 Karachalios T, Komnos G, Koutalos A. Total hip arthroplasty: survival and modes of failure. *EFORT open reviews* 2018;**3**:232–9.

29 Sayers AE, Deere KC, Lenguerrand E, *et al.* The COVID-19 induced joint replacement deficit in England, Wales and Northern Ireland. *National Joint Registry 18th Annual Report* Published Online First: 2021.https://www.ncbi.nlm.nih.gov/books/NBK576854/ (accessed 19 Sep 2022).

30 Royal College of Surgeons of England. Surgeons call for investment in surgical hubs as NHS waiting list hits record 5.3 million. 2021.https://www.rcseng.ac.uk/news-and-events/media-centre/press-releases/waiting-times-may-2021/ (accessed 17 Dec 2021).

31 Royal College of Surgeons of England. The case for surgical hubs. Published Online First: 2022.https://www.rcseng.ac.uk/about-the-rcs/government-relations-and-consultation/position-statements-and-reports/the-case-for-surgical-hubs/ (accessed 4 Oct 2022).

32 ODEP. Orthopaedic Data Evaluation Panel (ODEP). 2021.https://www.odep.org.uk/ (accessed 15 Apr 2021).

33 Abram SG, Sabah SA, Alvand A, *et al.* Differences in mortality and complication rates following revision knee arthroplasty performed for urgent versus elective indications. *The Bone & Joint Journal* 2021;**103**:1578–85. doi:10.1302/0301-620X.103B10.BJJ-2020-2590.R1

34 Boddapati V, Fu MC, Tetreault MW, *et al.* Short-term complications after revision hip arthroplasty for prosthetic joint infection are increased relative to noninfectious revisions. *The Journal of arthroplasty* 2018;**33**:2997–3002.

35 Sabah SA, Hedge EA, von Fritsch L, *et al.* Patient-relevant outcomes following elective, aseptic revision knee arthroplasty: a systematic review. *Systematic Reviews* 2023;**12**:133.

36 Sabah S, Knight R, Alvand A, *et al.* Patient-Relevant Outcomes Following First Revision Total Knee Arthroplasty, by Diagnosis: An Analysis of Implant Survivorship, Mortality, Serious Medical Complications, and Patient-Reported Outcome Measures Utilizing the National Joint Registry Data. *JBJS* 2023.

For peer review only

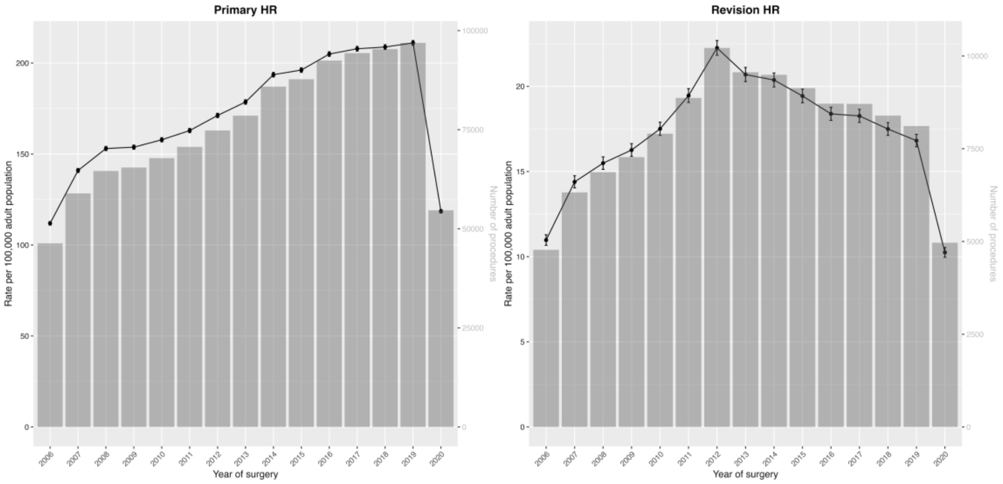

Figure 1: Annual crude incidence rates (line, left y-axis) and total counts (bars, right y-axis) of (a) pHR and (b) rHR from 2006-2020. Note the differences in y-axis scales for pHR and rHR

199x99mm (600 x 600 DPI)

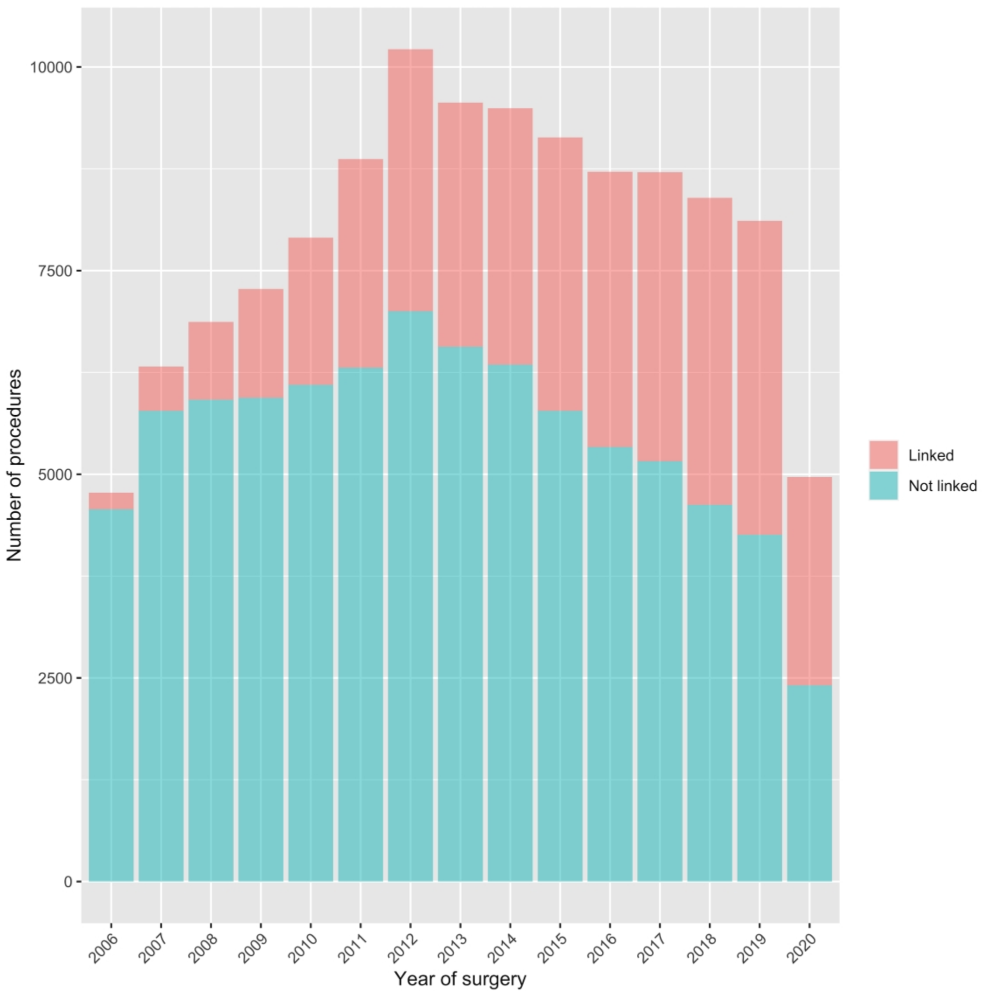

Figure 2: Changes in the annual total counts of rHR that could be linked to a pHR on the NJR over time

1000x1000mm (72 x 72 DPI)

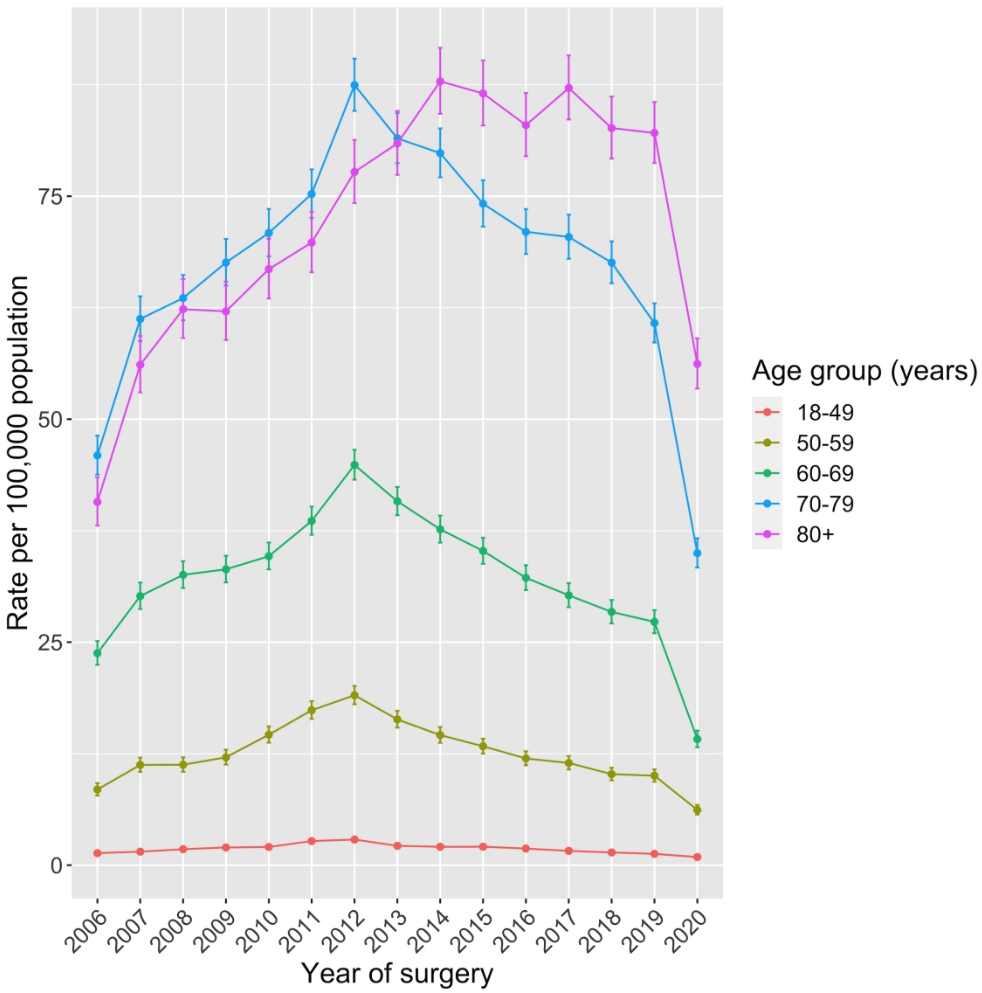

Figure 3: Annual incidence rates of first linked rHR from 2006-2020 by age group.

1000x1000mm (72 x 72 DPI)

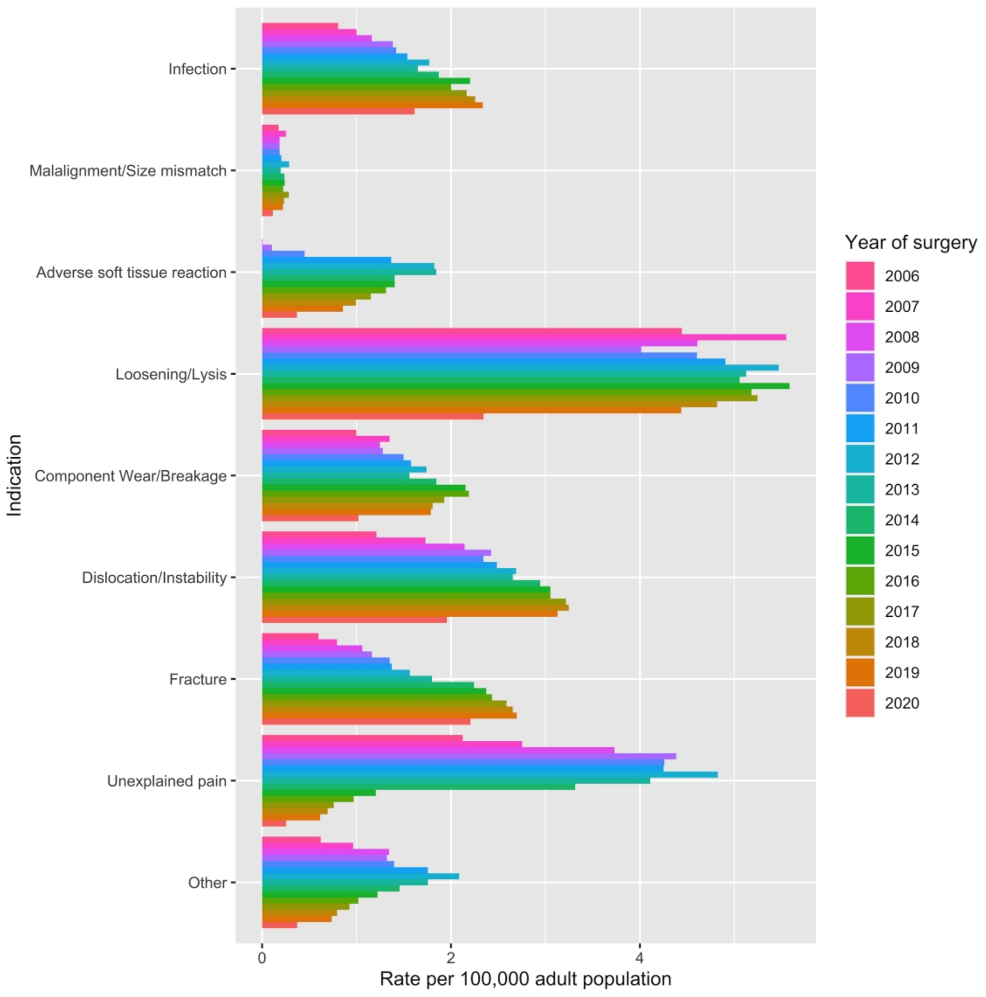

Figure 4: Grouped barplots demonstrating annual incidence rates for all rHR by indication for surgery from 2006-2020. Diagnoses are ranked in hierarchical order (greatest importance at the top).

1000x1000mm (72 x 72 DPI)

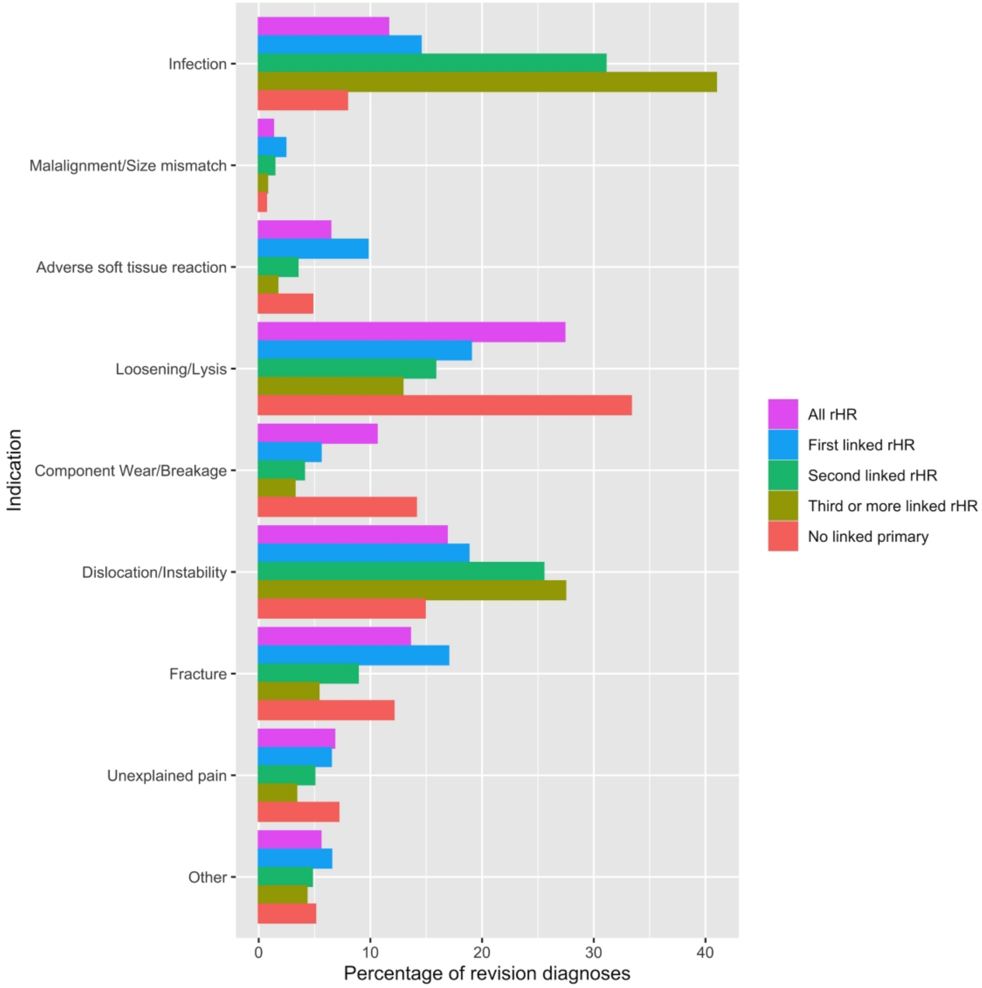

Figure 5: Percentage frequency of each indication for first and re-revision HR from 2014 to 2019. Diagnoses are ranked in hierarchical order (greatest importance at the top). For a given procedure (e.g. First linked rHR), the sum of all diagnoses is 100 percent.

1000x1000mm (72 x 72 DPI)

Supplementary Data

Appendix A - Methodology for calculation of directly standardised rates (DSRs) for pHR and rHR

Population estimates

The description that follows of the methodology for calculating directly standardised rates is reproduced from our recent publication [1].

We used mid-year population estimates from the Office for National Statistics (ONS) to calculate changes in the usual resident population. We filtered out population data for Scotland because it has its own register and does not report to the NJR. For simplicity, we have continued to refer to the data remaining (for England, Wales and Northern Ireland) as the 'UK population'. In addition, we have not accounted for population estimates for the Isle of Man and the States of Guernsey, which recently joined the NJR.

The total UK population increased in size every year over the study period, with overall growth of 10.1% from mid-2006 (55693967 persons) to mid-2019 (61333507 persons) [2]. The adult population (i.e. persons aged 18 years or older) increased by 10.9% from mid-2006 (43511312 persons) to mid-2019 (48239295 persons).

For peer review only

Methodology for population standardisation

We performed direct standardisation using Byar's method with Dobson method adjustment following Public Health England guidance [3]. The phe_dsr function within the PHEindicatormethods package in R was used. The 2013 European Standard Population (ESP) was chosen as the standard population. It is a theoretical population created from European Union states' population projections for 2011 - 2030. The population is divided into five-year age groups, starting from 0-4 years until 90 years and older. Since nearly all pHR and rHR are performed in adult patients, we truncated and recategorised the European Standard Population to match the age grouped we defined for the NJR (i.e. 18-49 years, 50-59 years, 60-69 years, 70-79 years, and 80+ years). To calculate the size of the 18-59 years age group, we calculated the total of all age groups from 20-24 years to 45-49 years, and then added two-fifths of the total of the 15-19 years age group. Finally, the resulting vector was transformed to provide a standard population of 100,000 persons.

Trends over time in directly standardised rates of pHR and rHR

Directly standardised rates of pHR and rHR are plotted together with crude incidence rates in Appendix A Figure 1. These rates allow other regions (including those with different age structures) to compare their rate of intervention to the UK.

For peer review only

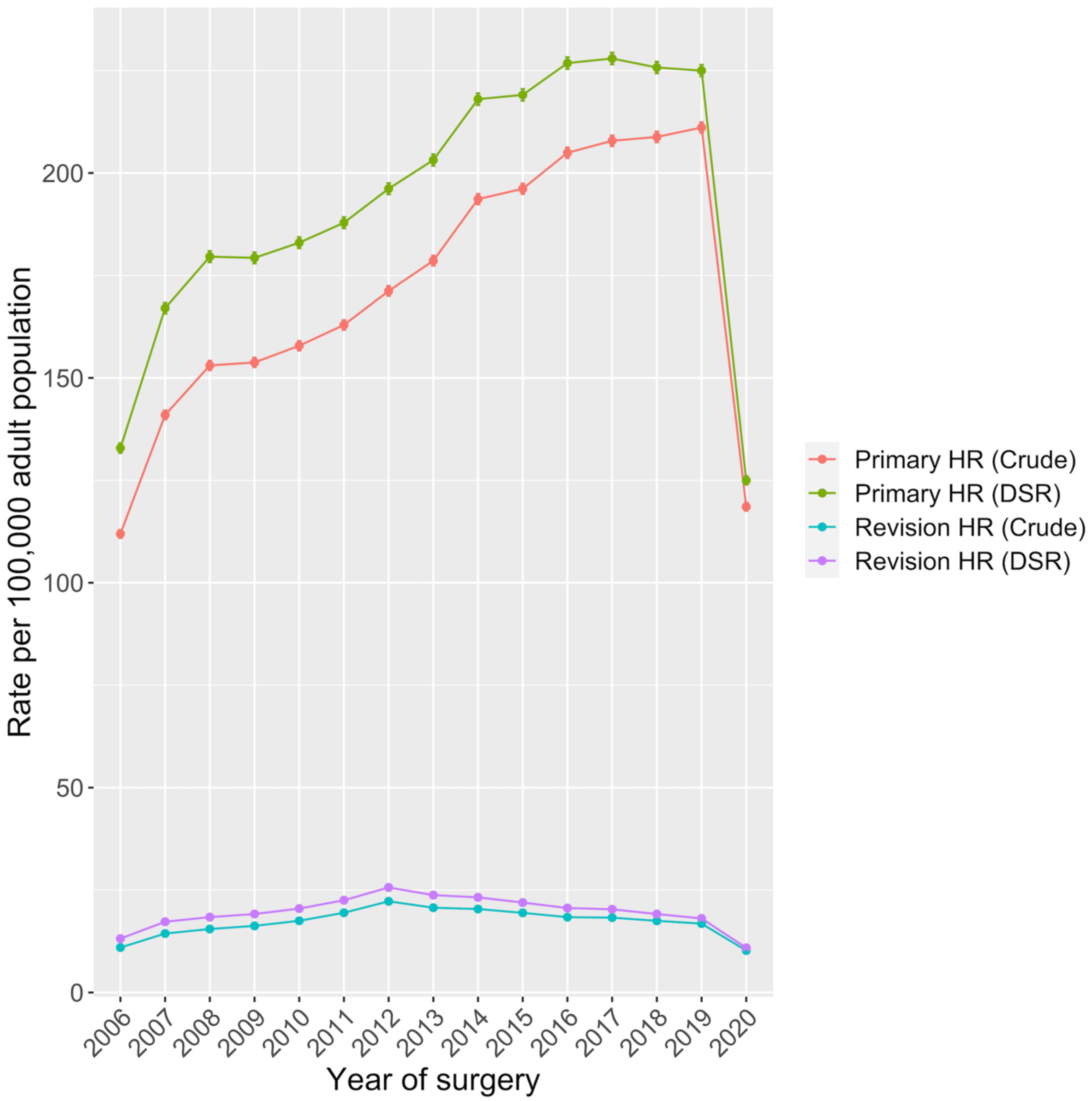

Appendix A Figure 1: Directly standardised rates (DSR) (using the European Standard Population 2013) and crude rates of pHR and rHR from 2006-2020.

Appendix B - Diagnosis hierarchy for rHR

Appendix B Table 1: Diagnosis hierarchy for rHR

Rank	Indication for revision	NJR indication(s) included	AOANJRR category
1	Infection	Infection	Dominant diagnosis independent of prosthesis/surgery
2	Malalignment/Size mismatch	Head/Socket Mismatch<br>Malalignment	Surgical procedure
3*	Adverse soft tissue reaction	Adverse Soft Tissue Reaction to Particulate Debris	Reaction to prosthesis
4	Loosening/Lysis	Aseptic loosening<br>Lysis	Reaction to prosthesis
5	Component wear/Breakage	Implant fracture<br>Wear of acetabular component	Wear and implant breakage
6	Dislocation/Instability	Dislocation/Subluxation<br>Dissocation of Liner	Stability of prosthesis
7	Fracture	Peri-Prosthetic Fracture	Fracture of bone
9	Unexplained pain	Unexplained pain	Pain
10	Other	Other	Remaining diagnoses

*Indication added to NJR in 2008

Appendix B Table 2: Counts and percentage frequency for each indication for rHR from 2006-2019 combined

Indication for revision	n	Percent
Infection	10938	9.6
Malalignment/Size mismatch	1433	1.3
Adverse soft tissue reaction	5940	5.2
Loosening/Lysis	31796	27.8
Component Wear/Breakage	10625	9.3
Dislocation/Instability	16830	14.7
Fracture	11507	10.1
Unexplained pain	17286	15.1
Other	7993	7.0

peer review only

Appendix C – Data Cleaning

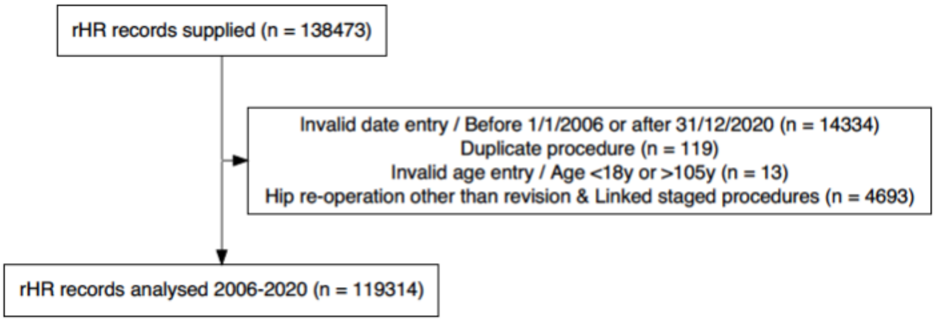

Appendix C Figure 1: Flowchart demonstrating attrition of study records during data cleaning.

For peer review only

Appendix D – Further analyses of trends over time

	2006	2007	2008	2009	2010	2011	2012	2013	2014	2015	2016	2017	2018	2019	2020
**Annual totals of rHR procedures**															
All rHR	4775	6322	6870	7274	7905	8869	10217	9562	9493	9134	8714	8709	8393	8111	4966
No linked primary	4570	5779	5915	5938	6098	6308	7001	6566	6346	5780	5334	5160	4624	4257	2408
First linked rHR	202	522	899	1234	1637	2309	2840	2634	2758	2896	2919	3044	3224	3297	2163
Second linked rHR	2	19	52	90	141	219	324	309	308	365	361	401	401	427	306
Third or more linked rHR	1	2	4	12	29	33	52	53	81	93	100	104	144	130	89
**Incidence rates of rHR**															
Crude incidence rate per 100,000 adults	11 (10.7 - 11.3)	14.4 (14 - 14.8)	15.5 (15.1 - 15.9)	16.3 (15.9 - 16.6)	17.5 (17.1 - 17.9)	19.5 (19.1 - 19.9)	22.3 (21.8 - 22.7)	20.7 (20.3 - 21.1)	20.4 (20 - 20.8)	19.4 (19 - 19.8)	18.4 (18 - 18.8)	18.3 (17.9 - 18.7)	17.5 (17.1 - 17.9)	16.8 (16.5 - 17.2)	10.2 (10 - 10.5)
Age-specific incidence rate per 100,000 persons															
18-49 years	1.3 (1.2 - 1.5)	1.5 (1.4 - 1.7)	1.8 (1.6 - 2)	2 (1.8 - 2.2)	2 (1.9 - 2.2)	2.7 (2.5 - 2.9)	2.9 (2.7 - 3.1)	2.2 (2 - 2.4)	2.1 (1.9 - 2.2)	2.1 (1.9 - 2.2)	1.9 (1.7 - 2)	1.6 (1.5 - 1.8)	1.4 (1.3 - 1.6)	1.3 (1.1 - 1.4)	0.9 (0.8 - 1)
50-59 years	8.5 (7.8 - 9.2)	11.2 (10.5 - 12.1)	11.3 (10.5 - 12.1)	12.1 (11.3 - 12.9)	14.6 (13.7 - 15.6)	17.4 (16.4 - 18.4)	19 (18 - 20.1)	16.3 (15.4 - 17.3)	14.6 (13.7 - 15.5)	13.3 (12.5 - 14.2)	12 (11.2 - 12.8)	11.5 (10.7 - 12.2)	10.2 (9.5 - 10.9)	10 (9.4 - 10.7)	6.2 (5.7 - 6.8)
60-69 years	23.8 (22.5 - 25.1)	30.2 (28.7 - 31.7)	32.5 (31.1 - 34.1)	33.2 (31.7 - 34.7)	34.6 (33.2 - 36.2)	38.6 (37 - 40.2)	44.9 (43.2 - 46.6)	40.8 (39.2 - 42.4)	37.7 (36.2 - 39.2)	35.2 (33.8 - 36.7)	32.2 (30.8 - 33.6)	30.3 (28.9 - 31.6)	28.4 (27.1 - 29.7)	27.3 (26 - 28.6)	14.1 (13.2 - 15.1)
70-79 years	45.9 (43.8 - 48.2)	61.2 (58.8 - 63.8)	63.6 (61.1 - 66.2)	67.6 (65 - 70.2)	70.9 (68.3 - 73.6)	75.3 (72.6 - 78)	87.5 (84.6 - 90.4)	81.5 (78.7 - 84.3)	79.8 (77.1 - 82.6)	74.2 (71.6 - 76.8)	71 (68.5 - 73.6)	70.4 (68 - 72.9)	67.6 (65.2 - 70)	60.8 (58.6 - 63)	35 (33.4 - 36.7)
80+ years	40.7 (38.1 - 43.5)	56.1 (53 - 59.3)	62.3 (59.1 - 65.7)	62.1 (58.9 - 65.4)	66.8 (63.5 - 70.2)	69.8 (66.5 - 73.3)	77.7 (74.2 - 81.3)	80.9 (77.4 - 84.6)	87.9 (84.2 - 91.6)	86.5 (82.9 - 90.2)	83 (79.5 - 86.6)	87.1 (83.6 - 90.8)	82.6 (79.2 - 86.2)	82.1 (78.7 - 85.6)	56.2 (53.4 - 59.1)

Appendix D Table 1: Annual totals and incidences rates of rHR

For peer review only - http://bmjopen.bmj.com/site/about/guidelines.xhtml

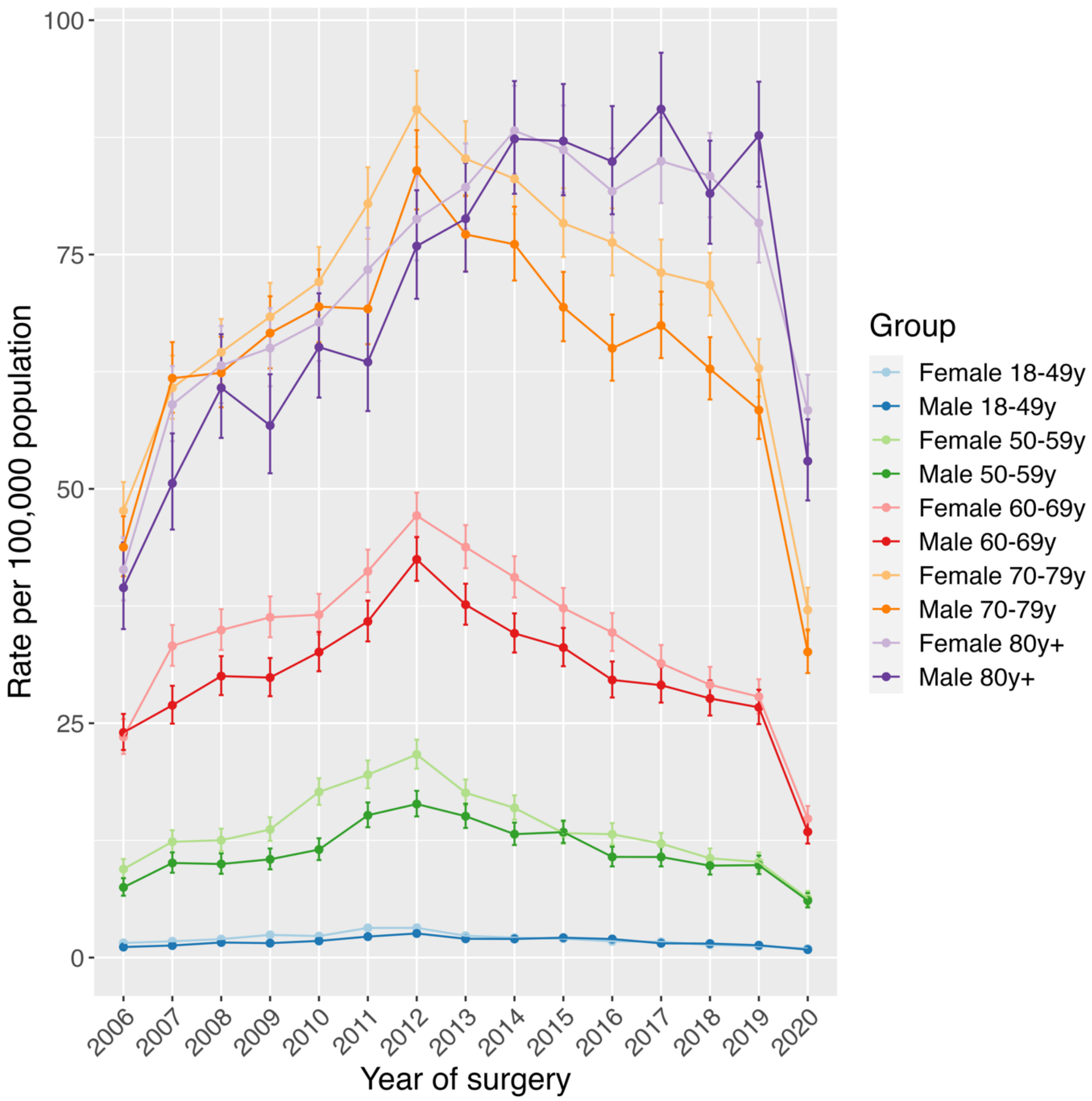

Appendix D Figure 1: Age- and gender-specific annual incidence rates for all rHR from 2006-2020. The rate of intervention was generally higher in female patients, though differences were smaller in more recent years.

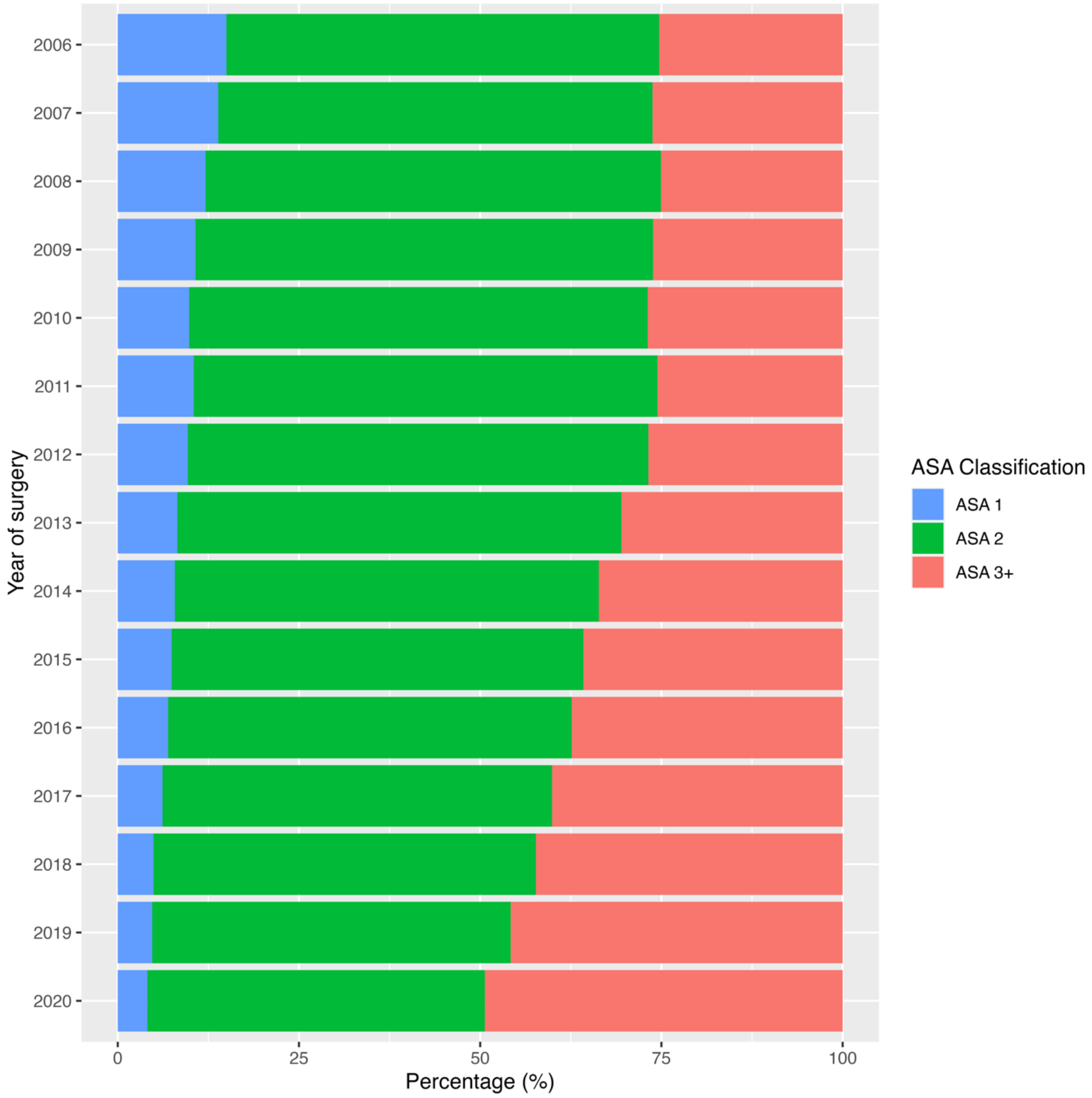

Appendix D Figure 2: Trends over time in the annual proportion of each American Society of Anesthesiologists (ASA) classification in patients undergoing all rHR between 2006-2020. The proportion of ASA Class 3+ patients increased from 25.3% in 2006 to 45.8% in 2019, with a further increase in 2020 (49.4%). Both ASA Class 1 and ASA Class 2 groups became smaller over the study period.

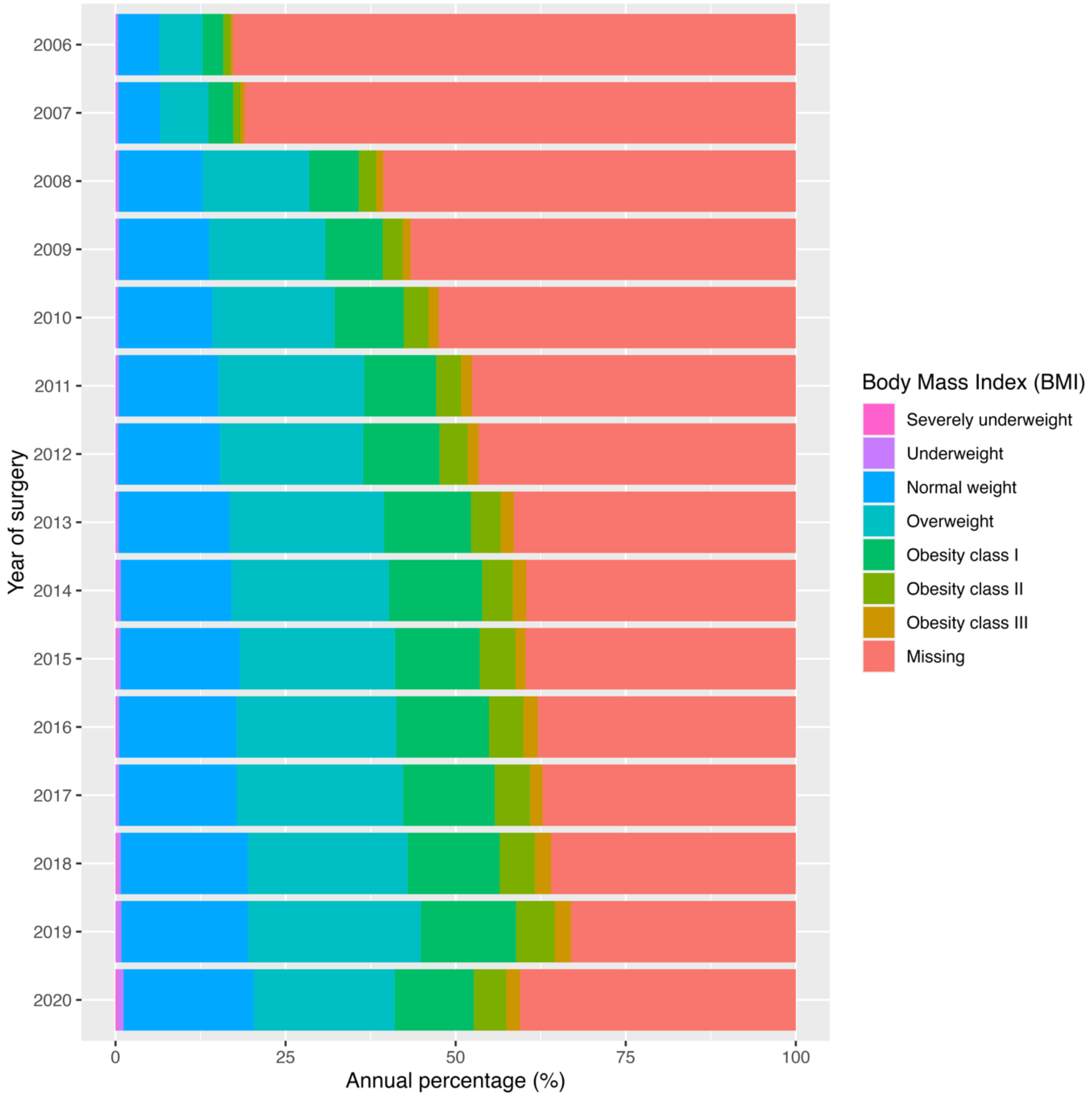

Appendix D Figure 3: Trends over time in the annual proportion of each Body Mass Index (BMI) classification in patients undergoing all rHR between 2006-2020. BMI was categorized following World Health Organisation cut-offs [4]. This figure demonstrates that BMI data were missing for a large proportion of procedures, with data collection improving over time. In 2006, 82.7% of records were missing BMI data, compared to 33.1% in 2019.

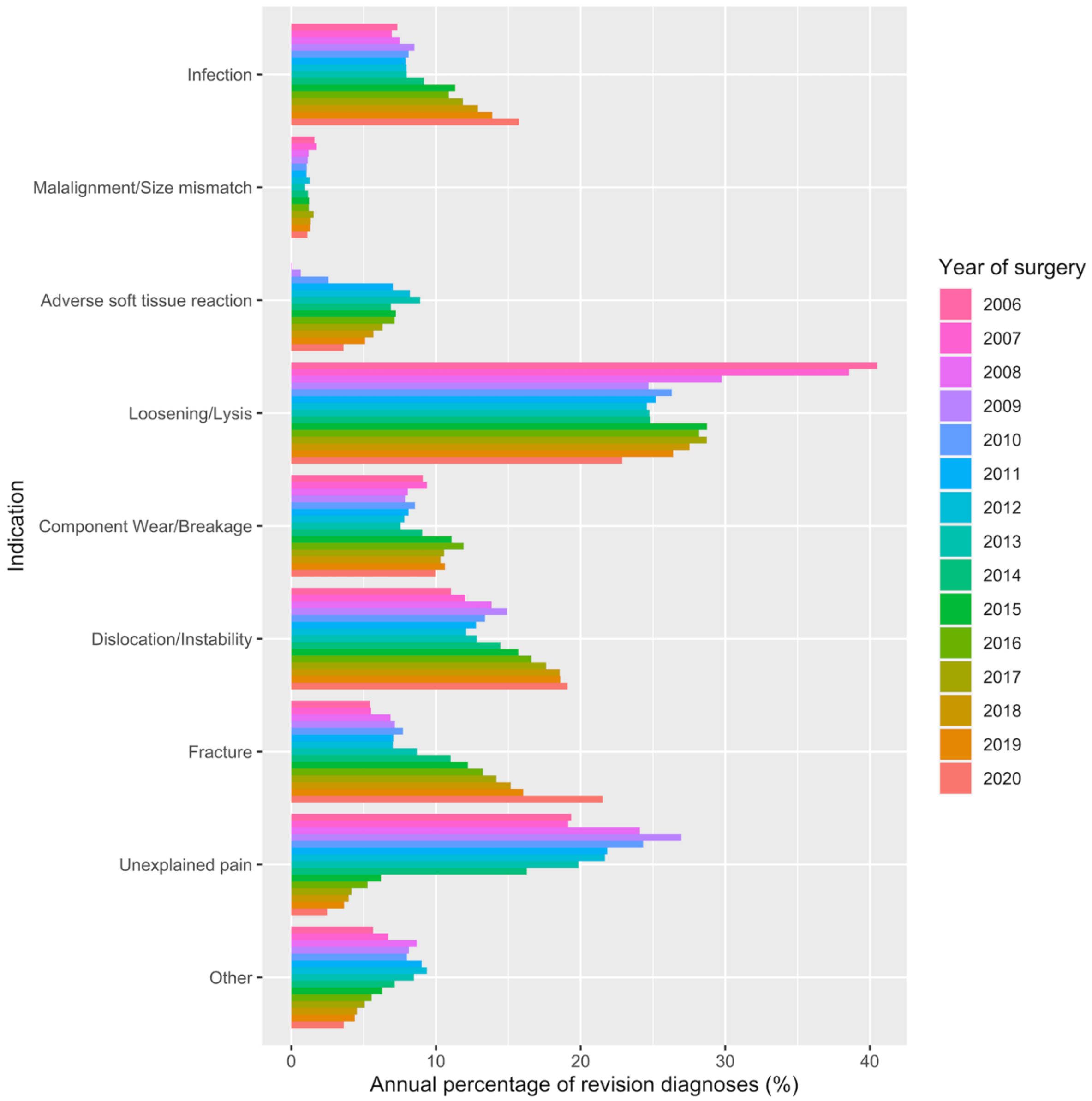

Appendix D Figure 4: Grouped barplots demonstrating changes in the annual proportions of each revision indication for all rHR from 2006-2020. Indications are ranked in hierarchical order (greatest importance at the top).

References

1. Sabah SA, Knight R, Alvand A, et al. No exponential rise in revision knee replacement surgery over the past 15 years: An analysis from the National Joint Registry. *Osteoarthritis and Cartilage*. Published online 2022. doi:10.1016/j.joca.2022.08.016

2. Office for National Statistics (ONS). Population estimates. Accessed November 19, 2021. https://www.ons.gov.uk/peoplepopulationandcommunity/populationandmigration/populationestimates

3. Public Health England. Technical guide: Confidence Intervals. Published online May 2018. Accessed November 19, 2021. https://fingertips.phe.org.uk/documents/PHDS%20Guidance%20-%20Confidence%20Intervals.pdf

4. Obesity: preventing and managing the global epidemic. Report of a WHO consultation. *World Health Organ Tech Rep Ser*. 2000;894:i-xii, 1-253.

For peer review only

The RECORD statement – checklist of items, extended from the STROBE statement, that should be reported in observational studies using routinely collected health data.

	Item No.	STROBE items	Location in manuscript where items are reported	RECORD items	Location in manuscript where items are reported
**Title and abstract**					
	1	(a) Indicate the study's design with a commonly used term in the title or the abstract (b) Provide in the abstract an informative and balanced summary of what was done and what was found		RECORD 1.1: The type of data used should be specified in the title or abstract. When possible, the name of the databases used should be included.<br><br>RECORD 1.2: If applicable, the geographic region and timeframe within which the study took place should be reported in the title or abstract.<br><br>RECORD 1.3: If linkage between databases was conducted for the study, this should be clearly stated in the title or abstract.	Title/Abstract<br><br>Abstract<br><br>Abstract
**Introduction**					
Background rationale	2	Explain the scientific background and rationale for the investigation being reported			L58-92
Objectives	3	State specific objectives, including any prespecified hypotheses			L91-92
**Methods**					
Study Design	4	Present key elements of study design early in the paper			Abstract & L94-204
Setting	5	Describe the setting, locations, and relevant dates, including periods of recruitment, exposure, follow-up, and data collection			L116-125

For peer review only

Participants	6	*(a) Cohort study* - Give the eligibility criteria, and the sources and methods of selection of participants. Describe methods of follow-up<br>*Case-control study* - Give the eligibility criteria, and the sources and methods of case ascertainment and control selection. Give the rationale for the choice of cases and controls<br>*Cross-sectional study* - Give the eligibility criteria, and the sources and methods of selection of participants<br><br>*(b) Cohort study* - For matched studies, give matching criteria and number of exposed and unexposed<br>*Case-control study* - For matched studies, give matching criteria and the number of controls per case		RECORD 6.1: The methods of study population selection (such as codes or algorithms used to identify subjects) should be listed in detail. If this is not possible, an explanation should be provided.<br><br>RECORD 6.2: Any validation studies of the codes or algorithms used to select the population should be referenced. If validation was conducted for this study and not published elsewhere, detailed methods and results should be provided.<br><br>RECORD 6.3: If the study involved linkage of databases, consider use of a flow diagram or other graphical display to demonstrate the data linkage process, including the number of individuals with linked data at each stage.	Population selection described L116-125<br><br>NJR validation studies referenced<br><br>Figure 6
Variables	7	Clearly define all outcomes, exposures, predictors, potential confounders, and effect modifiers. Give diagnostic criteria, if applicable.		RECORD 7.1: A complete list of codes and algorithms used to classify exposures, outcomes, confounders, and effect modifiers should be provided. If these cannot be reported, an explanation should be provided.	Ref 16.
Data sources/ measurement	8	For each variable of interest, give sources of data and details of methods of assessment (measurement).<br>Describe comparability of assessment methods if there is more than one group			L127-195

For peer review only

Bias	9	Describe any efforts to address potential sources of bias			N/A
Study size	10	Explain how the study size was arrived at			All available data used
Quantitative variables	11	Explain how quantitative variables were handled in the analyses. If applicable, describe which groupings were chosen, and why			L127-195
Statistical methods	12	(a) Describe all statistical methods, including those used to control for confounding<br>(b) Describe any methods used to examine subgroups and interactions<br>(c) Explain how missing data were addressed<br>(d) *Cohort study* - If applicable, explain how loss to follow-up was addressed<br>*Case-control study* - If applicable, explain how matching of cases and controls was addressed<br>*Cross-sectional study* - If applicable, describe analytical methods taking account of sampling strategy<br>(e) Describe any sensitivity analyses			L127-195
Data access and cleaning methods		..		RECORD 12.1: Authors should describe the extent to which the investigators had access to the database population used to create the study population.	See GitHub repo

For peer review only

				RECORD 12.2: Authors should provide information on the data cleaning methods used in the study.	
Linkage		..		RECORD 12.3: State whether the study included person-level, institutional-level, or other data linkage across two or more databases. The methods of linkage and methods of linkage quality evaluation should be provided.	Person-level<br>See GitHub repo
**Results**					
Participants	13	(a) Report the numbers of individuals at each stage of the study (*e.g.*, numbers potentially eligible, examined for eligibility, confirmed eligible, included in the study, completing follow-up, and analysed)<br>(b) Give reasons for non-participation at each stage.<br>(c) Consider use of a flow diagram		RECORD 13.1: Describe in detail the selection of the persons included in the study (*i.e.,* study population selection) including filtering based on data quality, data availability and linkage. The selection of included persons can be described in the text and/or by means of the study flow diagram.	Figure 6
Descriptive data	14	(a) Give characteristics of study participants (*e.g.*, demographic, clinical, social) and information on exposures and potential confounders<br>(b) Indicate the number of participants with missing data for each variable of interest<br>(c) *Cohort study* - summarise follow-up time (*e.g.*, average and total amount)			Mostly N/A<br><br>However, see Table 1
Outcome data	15	*Cohort study* - Report numbers of outcome events or summary measures over time<br>*Case-control study* - Report numbers in each exposure			N/A

For peer review only

		category, or summary measures of exposure<br>*Cross-sectional study* - Report numbers of outcome events or summary measures			
Main results	16	(a) Give unadjusted estimates and, if applicable, confounder-adjusted estimates and their precision (e.g., 95% confidence interval). Make clear which confounders were adjusted for and why they were included<br>(b) Report category boundaries when continuous variables were categorized<br>(c) If relevant, consider translating estimates of relative risk into absolute risk for a meaningful time period			N/A
Other analyses	17	Report other analyses done—e.g., analyses of subgroups and interactions, and sensitivity analyses			
**Discussion**					
Key results	18	Summarise key results with reference to study objectives			L270-284
Limitations	19	Discuss limitations of the study, taking into account sources of potential bias or imprecision. Discuss both direction and magnitude of any potential bias		RECORD 19.1: Discuss the implications of using data that were not created or collected to answer the specific research question(s). Include discussion of misclassification bias, unmeasured confounding, missing data, and changing eligibility over time, as they pertain to the study being reported.	L307-326
Interpretation	20	Give a cautious overall interpretation of results considering objectives,			L328-337

		limitations, multiplicity of analyses, results from similar studies, and other relevant evidence			
Generalisability	21	Discuss the generalisability (external validity) of the study results			L328-337
**Other Information**					
Funding	22	Give the source of funding and the role of the funders for the present study and, if applicable, for the original study on which the present article is based			Title page
Accessibility of protocol, raw data, and programming code		..		RECORD 22.1: Authors should provide information on how to access any supplemental information such as the study protocol, raw data, or programming code.	GitHub repository

*Reference: Benchimol EI, Smeeth L, Guttmann A, Harron K, Moher D, Petersen I, Sørensen HT, von Elm E, Langan SM, the RECORD Working Committee. The REporting of studies Conducted using Observational Routinely-collected health Data (RECORD) Statement. *PLoS Medicine* 2015; in press.

*Checklist is protected under Creative Commons Attribution (CC BY) license.

For peer review only - http://bmjopen.bmj.com/site/about/guidelines.xhtml
